# Modeling enzyme competition in eicosanoid metabolism in macrophage cells using a cybernetic framework

**DOI:** 10.1016/j.jlr.2024.100666

**Published:** 2024-10-11

**Authors:** Sana Khanum, Shakti Gupta, Mano R. Maurya, Rubesh Raja, Lina Aboulmouna, Shankar Subramaniam, Doraiswami Ramkrishna

**Affiliations:** 1 The Davidson School of Chemical Engineering, Purdue University, West Lafayette, IN, USA; 2 Department of Bioengineering, University of California San Diego, La Jolla, CA, USA; 3 Departments of Computer Science and Engineering, Cellular and Molecular Medicine, San Diego Supercomputer Center, and the Graduate Program in Bioinformatics and Systems Biology, University of California San Diego, La Jolla, CA, USA

**Keywords:** arachidonic acid, cyclooxygenase, inflammation, lipidomics, lipolysis and fatty acid metabolism, omega-3 fatty acid, prostaglandin, eicosapentaenoic acid, kinetic modeling

## Abstract

Cellular metabolism is a complex process involving the consumption and production of metabolites, as well as the regulation of enzyme synthesis and activity. Modeling of metabolic processes is important to understand the underlying mechanisms, with a wide range of applications in metabolic engineering and health sciences. Cybernetic modeling is a powerful technique that accounts for unknown intricate regulatory mechanisms in complex cellular processes. It models regulation as goal-oriented, where the levels and activities of enzymes are modulated by the cybernetic control variables to achieve the cybernetic objective. This study used cybernetic model to study the enzyme competition between arachidonic acid (AA) and eicosapentaenoic acid (EPA) metabolism in murine macrophages. AA and EPA compete for the shared enzyme cyclooxygenase. Upon external stimuli, AA produces proinflammatory 2-series prostaglandins and EPA metabolizes to antiinflammatory 3-series prostaglandins, where proinflammatory and antiinflammatory responses are necessary for homeostasis. The cybernetic model adequately captured the experimental data for control and EPA-supplemented conditions. The model is validated by performing an F-test, conducting leave-one-out-metabolite cross-validation, and predicting an unseen experimental condition. The cybernetic variables provide insights into the competition between AA and EPA for the cyclooxygenase enzyme. Predictions from our model suggest that the system undergoes a switch from a predominantly proinflammatory state in the control to an antiinflammatory state with EPA-supplementation. The model can also be used to analytically determine the AA and EPA concentrations required for the switch to occur. The quantitative outcomes enhance understanding of proinflammatory and antiinflammatory metabolism in RAW 264.7 macrophages.

A metabolic system consists of a cascade of chemical reactions that result in the production of diverse metabolites, facilitated by specific enzymes acting on substrates. A comprehensive understanding of metabolic processes is essential for implementing genetic modifications to enhance metabolic performance as well as control biological processes (1, 2). Quantitative modeling of these metabolic processes can be a valuable tool in achieving these objectives (3, 4, 5, 6, 7, 8, 9, 10, 11, 12). In cellular metabolism, enzymes are regulated by various mechanisms, including synthesis and modulation of their activity. Mechanistic insights can be gained from models such as flux balance analysis, and kinetic models such as the Michaelis-Menten formulation, adapted for various scenarios including ping-pong kinetics, sequential kinetics, and competitive or noncompetitive enzyme inhibition (9, 13, 14, 15, 16, 17, 18, 19, 20, 21, 22, 23, 24). However, these models may not fully incorporate the complex regulatory mechanisms as the specific details of these regulatory processes are often not well understood, which makes it challenging to incorporate them into the models (25, 26, 27, 28). The cybernetic modeling approach, a technique used for modeling of cellular metabolic processes, addresses this limitation by defining a biological goal that the system aims to optimize. It also takes into account the dynamics of enzymes, which allows the model to predict enzyme profiles necessary for metabolism, even in cases where enzyme measurements are unavailable (25, 26, 27). The biological goal is formulated as the cybernetic goal and refers to the maximization of the sum of fluxes associated with selected metabolites/processes. The model assumes that the system achieves the formulated goal by adjusting the levels and activities of the enzymes involved in the respective reactions, and the reactions compete for available resources necessary for metabolic transformations. To represent this competition, the cybernetic model introduces cybernetic control variables, denoted as *u* and *v*, for the participating metabolites. The variable *u* represents the control variable for the enzyme synthesis process and allocates resources for metabolic conversions in the competing reactions. The cybernetic variable *v* regulates the activity of the corresponding enzyme. These control variables consider both known and unknown regulatory steps and modulate the competing reactions to achieve the specified biological goal (25, 26, 27). The cybernetic framework has undergone progressive evolution, surpassing its initial limitations and emerging as a promising approach to comprehensively and quantitatively describe metabolism. Cybernetic models have been successfully applied to describe various biological phenomena in unicellular organisms such as yeast and bacteria cells (29, 30). More recently, cybernetic models have also been used to study the inflammatory responses in mammalian cells (28, 31).

Arachidonic acid (AA) is an omega-6 PUFA, which produces proinflammatory 2-series prostaglandins (PGs) through the action of the cyclooxygenase (COX) enzyme. These 2-series PGs initiate acute inflammation and mediate pain and other symptoms during the inflammatory response (32, 33, 34). In contrast, eicosapentaenoic acid (EPA) and docosahexaenoic acid (DHA), which are omega-3 PUFAs, form antiinflammatory metabolites (35, 36, 37, 38). While humans can synthesize EPA and DHA from α-linolenic acid to some extent, it is necessary to supplement them through dietary sources such as fish, nutraceuticals, and functional foods (38, 39, 40, 41). Consumption of fish or fish oil, which is a significant source of EPA and DHA, reduces the production of proinflammatory 2-series PGs (38, 42, 43). This occurs because EPA and DHA compete with AA by (1) replacing it in the cell membrane phospholipid bilayer, thereby reducing the availability of AA for metabolism, and (2) sharing the COX enzyme. EPA and DHA are metabolized by COX to produce 3-series PGs. Unlike the proinflammatory AA-derived mediators, the 3-series PGs exhibit antiinflammatory actions by decreasing leukocyte chemotaxis, reactive oxygen species levels, and proinflammatory cytokine secretion (44, 45, 46). They also reduce adhesion molecule expression and inhibit platelet aggregation (44, 45). The production of proinflammatory lipid mediators from AA and antiinflammatory lipid mediators from EPA metabolism plays a critical role in initiating, progressing, and resolving the inflammatory response. Dysregulation of these processes can lead to chronic inflammation, tissue damage, and impaired healing (33, 34, 47, 48, 49). Excessive proinflammatory response contributes to the severity and progression of various diseases, including cancer and coronavirus disease 2019 (COVID-19) (50, 51). The antiinflammatory properties of lipid mediators make them potential therapeutic agents for these inflammatory conditions. Research suggests that the antiinflammatory nature of EPA and DHA can be beneficial in reducing the severity of diseases such as cancer, cardiovascular effects, and promoting visual and neurological development (37, 52, 53, 54). Nonsteroidal antiinflammatory drugs, which inhibit COX, are commonly used pharmacotherapeutic agents for treating inflammation. They exhibit antiinflammatory effects and help to block and alleviate inflammation (31, 32, 33, 34, 35, 36, 37). Studies involving humans have reported reduced mortality rates in patients with diseases due to increased intake of EPA-containing diets (55).

Computational kinetic models have proven to be valuable tools for understanding the underlying principles and uncovering novel mechanisms by capturing experimental data. In previous studies, linear or Michaelis-Menten kinetics were commonly used to capture the dynamic measurements of eicosanoid production resulting from AA metabolism (9, 20, 21, 22, 23, 24). While some experimental studies have investigated the metabolic analysis of AA profiles upon EPA/DHA supplementation (56, 57, 58, 59), fewer studies have focused on modeling the reaction network involving EPA/DHA. Norris and Dennis conducted an experimental study revealing a decrease in 2-series PGs and an increase in 3-series PGs in RAW 264.7 macrophages upon EPA and DHA supplementation (59). The observed phenomenon was attributed to AA and EPA/DHA competition for the enzyme COX. Gupta *et al.* developed a kinetic model using Michaelis-Menten dynamics to describe the competitive metabolism between AA and EPA/DHA (21).While their model reasonably captured general trends and saturating effects, it failed to accurately predict specific data points that deviated from the Michaelis-Menten behavior. One limitation of the Gupta *et al.* model was the noninclusion of mechanisms that are associated with metabolic regulation such as transcriptomic, translational, and posttranslational regulation and modifications. Our study suggests that usage of the cybernetic model can improve their model accuracy by incorporating known and unknown regulatory processes. Previous applications of cybernetic models have primarily focused on studying the dynamics of eicosanoids produced from the metabolism of AA during the inflammatory response of mammalian cells. This study aims to develop a cybernetic model for the reaction networks involving AA and EPA/DHA.

We developed a cybernetic model to study the AA and EPA/DHA metabolism in RAW 264.7 macrophage cells during the inflammatory response. AA and EPA/DHA share the enzyme COX to form proinflammatory 2-series and antiinflammatory 3-series PGs, respectively (21, 59). A recent study by Zaid *et al.* (60) observed high levels of both series PGs in intubated COVID-19 patients, emphasizing the critical roles of both stages of inflammation. Building upon these findings, we hypothesized that the biological goal during the inflammatory response is to maximize the combined proinflammatory and antiinflammatory responses in physiology. The cybernetic goal for AA and EPA/DHA metabolism is formulated to maximize AA and EPA/DHA consumption rates, as they are precursors of 2-series and 3-series PGs, respectively. The measurements are available for three scenarios: (1) the control: nonsupplemented, (2) EPA-supplemented, and (3) DHA-supplemented cases (59). The EPA-supplemented case was used to train the model, and it demonstrated reasonable agreement with the experimental data. It effectively captured the competitive nature between AA and EPA for the shared COX enzyme, represented as $e_{COX}$. We also validated the model by (1) performing an F-test to evaluate the goodness of fits, (2) performing leave-one-out-metabolite PGD_2_ cross-validation, and (3) predicting an unseen case of DHA supplementation. The predictions of enzyme profiles are realistic and corroborate the dynamics observed in the metabolite profiles. The dynamics of the cybernetic control variables effectively reflect the prevailing proinflammatory or antiinflammatory conditions observed in the experimental datasets.

## Materials and Methods

**Fig. 1:**
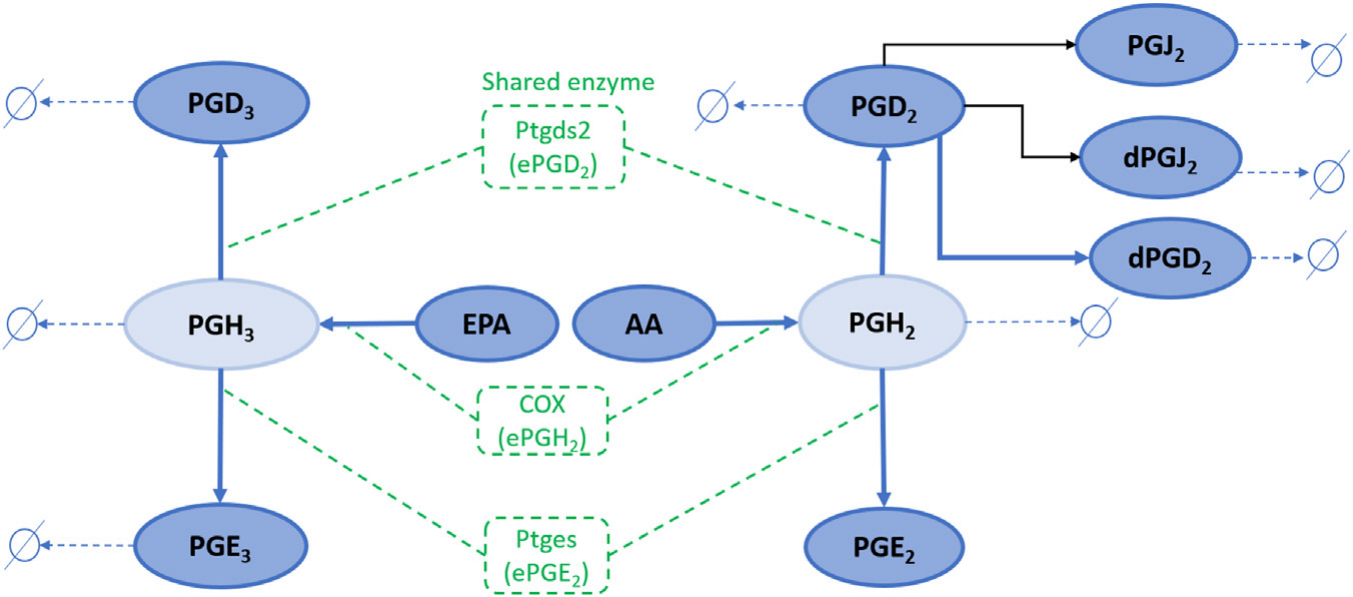
The arachidonic acid (AA) and eicosapentaenoic acid (EPA) reaction network. AA and EPA metabolism leads to 2-series and 3-series PGs, respectively. The 2-series PGs include PGD_2_, PGE_2_, dPGD2, and PGJ_2_. PGD_3_ and PGE_3_ are 3-series PGs. Darker blue ovals represent measured metabolites. Light blue ovals represent unmeasured intermediates, PGH_2_ and PGH_3_. Green-colored dashed boxes represent enzymes. The COX enzyme ($e_{COX}$) metabolizes AA and EPA. Similarly, $e_{Ptges}$, $e_{Ptgds}$ catalyze the conversion of PGH_2_ and PGH_3_ into downstream products. COX, cyclooxygenase; PG, prostaglandin.

**Fig. 2:**
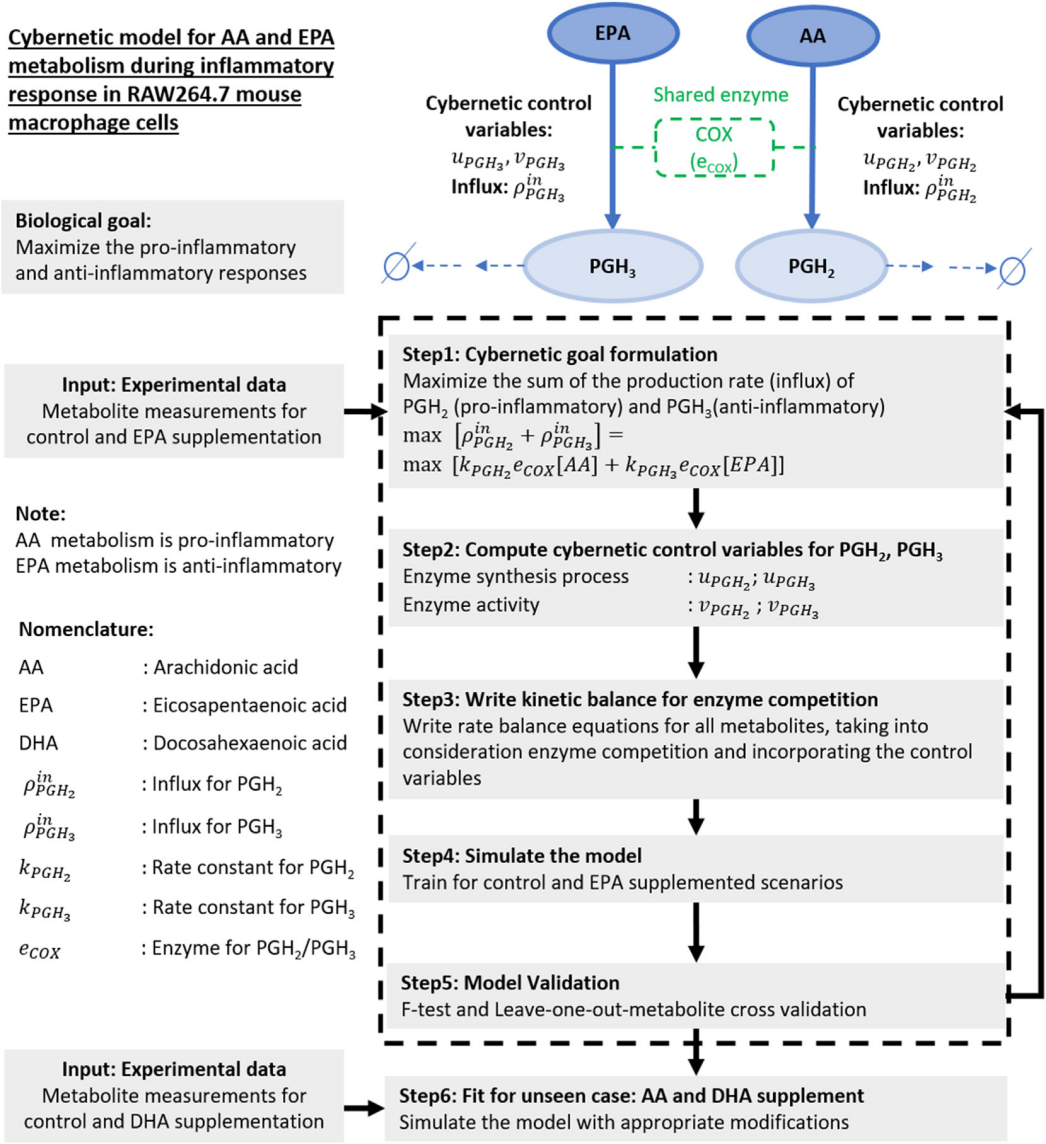
The schematic demonstrates the mathematical framework of the cybernetic model for AA and EPA metabolism in mammalian cells. The cybernetic control is introduced to the conversion of AA and EPA, leading to PGH_2_ and PGH_3_, respectively. A portion of the reaction network is shown on the top right. The biological goal is hypothesized to maximize the proinflammatory (exhibited by AA metabolism) and antiinflammatory (characteristic of EPA metabolism) responses. The model development includes formulating the cybernetic goal (step 1), which is the mathematical form of the biological goal, computing the cybernetic control variables (step 2), writing the rate balance forms for metabolites and enzymes (step 3), and training for the EPA supplemented case (step 4) and validating (step 5) the model by performing F-test and leave-one-out-metabolite cross-validation method. The final step is applying the developed model to an unseen DHA supplementation case with suitable modifications. AA, arachidonic acid; DHA, docosahexaenoic acid; EPA, eicosapentaenoic acid.

The reaction network for AA and EPA metabolism is shown in Fig. 1. AA metabolism produces PGH_2_, PGE_2_, PGD_2_, 15d-PGD_2_, PGJ_2_, and DHK-PGD_2_. EPA metabolism forms PGH_3_, PGE_3_, and PGD_3_. The measurements for PGH_2_ and PGH_3_ are not available because of their highly unstable structure. The experimental data on ATP activated RAW 264.5 is available for three conditions (1) control nonsupplemented, (2) EPA supplemented, and (3) DHA supplemented (59). The measurements are made at 0, 2.5, 5, 10, 15, 30, and 60 min post ATP stimulation. The control scenario has basal level of AA, and for EPA (DHA)-supplemented case, EPA (DHA) is added 24 h before ATP stimulation. ATP is a danger signal and an inflammatory stimulus. It activates cytosolic phospholipase A2, producing free AA through hydrolysis of AA esterified in membrane phospholipids (61). The 2-series PGs, including PGE_2_, PGD_2_, 15d-PGD_2_, PGJ_2_, and DHK-PGD_2_, are formed due to AA metabolism. The data for the 2-series PGs are measured for the three experimental conditions mentioned above. The data for the 3-series PGs are measured for the control and EPA-supplemented scenarios. In the nonsupplemented (control) condition, the low levels of EPA prevent significant conversion to 3-series PGs, resulting in negligible levels of these metabolites in the control scenario. However, upon supplementation with EPA or DHA, increased levels of EPA or DHA, respectively, are observed. ATP stimulation triggers EPA metabolism to produce 3-series PGs, comprising PGE_3_ and PGD_3_ (44). The DHA metabolism includes the product electrophile oxo-derivatives (EFOXs) due to the action of the COX enzyme, but the EFOXs measurements are not available for control and DHA-supplemented condition (62). Moreover, Fig. 2 demonstrates the mathematical framework of the cybernetic model for AA and EPA metabolism in mammalian cells, which is discussed in detail subsequently.

### Cybernetic goal formulation

The cybernetic model assumes a cybernetic goal, which is the mathematical representation of the defined biological goal. The cybernetic goal aims to maximize the cumulative fluxes of specific metabolites that effectively reflect the desired biological goal. The reaction fluxes of these selected set of n reactions compete to maximize their individual contributions to the cybernetic goal. The cybernetic goal is formulated as Equation (13), (14), (15), (16).

(1) $$\max\;\sum_{i=1}^{n}\rho \_i\;$$

where $\rho_{i}$ is the reaction flux for each of these n metabolites. In this study, the cybernetic goal for AA and EPA metabolic network is hypothesized as maximizing the sum of AA and EPA consumption rates. PGH_2_ and PGH_3_ are chosen for the cybernetic goal formulation because they are the precursors of the proinflammatory and antiinflammatory conversions of AA and EPA, respectively. Consequently, the cybernetic goal is to maximize the sum of production rates of PGH_2_ and PGH_3_, respectively (Equations 2 and 3). This is true because our network includes the conversion of AA and EPA to PGH_2_ and PGH_3_ only, respectively. We denote the outflux (consumption rate) of AA and EPA by $\rho_{AA}^{out}$ and $\rho_{EPA}^{out}$, respectively (Equation 2). Similarly, the influx (production rate) of PGH_2_ is denoted by $\rho_{PGH_{2}}^{in}$, and PGH_3_ by $\rho_{PGH_{3}}^{in}$. The definitions of $\rho_{PGH_{2}}^{in}$ and $\rho_{PGH_{3}}^{in}$ are shown in (Equation 3). The cybernetic goal is formulated as follows (Equation 2). [.] represents the concentration.

(2) $$\max\;\rho_{AA}^{out}+\rho_{EPA}^{out}=\max[\rho_{{PGH}_{2}}^{in}+\rho_{{PGH}_{3}}^{in}]$$

where,

(3) $$\rho_{{PGH}_{2}}^{in}=k_{{PGH}_{2}}\left[e_{COX}\right]\left[AA\right];\;\rho_{{PGH}_{3}}^{in}=k_{{PGH}_{3}}\left[e_{COX}\right]\left[EPA\right]$$

### Cybernetic control variables computation

The computation of cybernetic control variables requires the cybernetic goal formulation. The control variables are defined using the matching and proportion laws (25, 27). The variable $v_{i}$ modulates the activity of the enzyme catalyzing the formation of $P_{i}$. Similarly, $u_{i}$ is responsible for controlling the synthesis process of the associated enzyme. $v_{i}$ and $u_{i}$ are defined for all enzymes involved in n reactions considered for the cybernetic goal. $v_{i}$ is proportional to the related reaction flux, $\rho_{i}$. It attains a maximum value of one for the reaction with the highest flux. $v_{i}=1$ indicates the enzyme is fully active for the corresponding reaction. $u_{i}$ is also proportional to reaction flux as more enzyme control is required for reactions with higher flux ($u_{i}$ <= 1). Hence, the sum of $u_{i}$ for competing reactions (number of reactions: n) sum to one. As a consequence, $v_{i}$ and $u_{i}$ are defined as follows (Equation 4):

(4) $$v_{i}=\frac{\rho_{i}}{\max_{j}\rho_{j}}\;;\;u_{i}=\frac{\rho_{i}}{\sum_{j=1}^{n}\rho_{j}}$$

Employing Equation 4 for n = 2, the control variables for PGH_2_ ($v_{PGH_{2}},u_{PGH_{2}}$) and PGH_3_ ($v_{PGH_{3}},u_{PGH_{3}}$) are defined as Equations 5 and 6.

(5) $$v_{{PGH}_{2}}=\frac{\rho_{{PGH}_{2}}^{in}}{\max\left(\rho_{{PGH}_{2}}^{in},\rho_{{PGH}_{3}}^{in}\right)}\;;\;v_{{PGH}_{3}}=\frac{\rho_{{PGH}_{3}}^{in}}{\max\left(\rho_{{PGH}_{2}}^{in},\rho_{{PGH}_{3}}^{in}\right)}$$

(6) $$u_{{PGH}_{2}}=\frac{\rho_{{PGH}_{2}}^{in}}{\rho_{{PGH}_{2}}^{in}+\rho_{{PGH}_{3}}^{in}}\;;\;u_{{PGH}_{3}}=\frac{\rho_{{PGH}_{3}}^{in}}{\rho_{{PGH}_{2}}^{in}+\rho_{{PGH}_{3}}^{in}}$$

### Kinetic rate balance incorporating cybernetic control for EPA-supplemented case

This section describes the rate balance of all reactions included in the network (Fig. 1). The enzymatic reactions are represented by Equation 7.

(7) $$S_{i}+e_{i}\overset{k_{P_{i}}}{\to }\;P_{i}$$

where $S_{i}$ is the substrate. The enzymes are denoted by $e_{i}$, and the products by $P_{i}$, which are formed due to the action of the enzyme $e_{i}$ on the substrate $S_{i}$. $k_{P_{i}}$ indicates the rate constant of the reaction producing $P_{i}$. In our study, the substrates $S_{i}$ for enzymatic reactions are AA, EPA, PGH_2_, PGH_3_, and PGD_2_. The enzymes $e_{i}$ are $e_{COX}$, $e_{Ptgds}$, and $e_{Ptges}$. The products $P_{i}$ are PGH_2_, PGH_3_, PGD_2_, PGD_3_, PGE_2_, PGE_3_, and dhkPGD_2_. We refer to supplemental section 1 for the full set of equations.

#### Enzymatic reactions with cybernetic control variables

As discussed, AA and EPA utilize the enzyme, COX, and COX levels are represented as $e_{COX}$. The chemical reaction of AA (Equation 8) and EPA (Equation 9) with $e_{COX}$ leads to the formation of products PGH_2_ and PGH_3_ with rate constants $k_{PGH_{2}}$ and $k_{PGH_{3}}$, respectively.

(8) $$AA+e_{COX}\overset{k_{PGH_{2}}}{\to }\;PGH_{2}$$

(9) $$EPA+e_{COX}\overset{k_{PGH_{3}}}{\to }\;PGH_{3}$$

The measurements of AA and EPA are input to the model. The generic form of the differential equation for enzymatic reactions with cybernetic variables is provided below in Equation 10.

(10) $$\frac{d\left[P_{i}\right]}{dt}=v_{P_{i}}k_{P_{i}}\left[S_{i}\right]\left[e_{i}\right]-g_{P_{i}}\left[P_{i}\right]-downstream\;fluxes$$

The first term denotes the production rate of $P_{i}$, and $v_{P_{i}}$ is the cybernetic control variable for enzyme activity of $e_{i}$. $g_{P_{i}}$ is the degradation rate. The downstream fluxes account for the subsequent metabolism of $P_{i}$. The kinetic rate balances of PGH_2_ (Equation 11) and PGH_3_ (Equation 12) are formulated using Equation 10, and they also include $\left(1+k_{ATP}\left[ATP\right]\right)$ in the first term.

(11) $$\frac{d\left[PGH_{2}\right]}{dt}=v_{PGH_{2}}k_{PGH_{2}}\left[AA\right][e_{COX}]\left(1+k_{ATP}\left[ATP\right]\right)-g_{PGH_{2}}[PGH_{2}]-k_{PGD_{2}}[PGH_{2}][e_{Ptgds}]-k_{PGE_{2}}[PGH_{2}][e_{Ptges}]$$

(12) $$\frac{d\left[PGH_{3}\right]}{dt}=k_{PGH_{3}}v_{PGH_{3}}\left[EPA\right][e_{COX}]\left(1+k_{ATP}\left[ATP\right]\right)-g_{PGH_{3}}[PGH_{3}]-k_{PGD_{3}}\left[PGH_{3}\right][e_{Ptgds}]-k_{PGE_{3}}\left[PGH_{3}\right][e_{Ptges}]$$

[.] represents the time-varying concentrations. The first term in Equations 11 and 12 is the rate of generation of PGH_2_ and PGH_3_, from the substrates AA and EPA, respectively, catalyzed by COX. In the expression $1+k_{ATP}\left[ATP\right]$, the first term captures the basal activity (for both enzymes COX1 and COX2), and the second term captures the increase in activity due to ATP stimulation on COX2 (inducible form). The cybernetic control variables are defined for AA and EPA metabolism reactions, forming PGH_2_ and PGH_3_, respectively. $v_{PGH_{2}}$ is the activity control variable for PGH_2_, and $v_{PGH_{3}}$ is for PGH_3_. Although the enzyme $e_{COX}$ is the same for AA and EPA, its activity can differ because it can bind with varying affinities to distinct substrates. $g_{PGH_{2}}$ and $g_{PGH_{3}}$ are the corresponding decay rates of PGH_2_ and PGH_3_. The downstream fluxes for PGH_2_ and PGH_3_ are due to their subsequent conversions, described by Equations (13), (14), (15), (16), which are enzymatic reactions without cybernetic control variables. The products PGD_2_ (Equation 13) and PGD_3_ (Equation 14) also share the enzyme $e_{Ptgds}$, and PGE_2_ (Equation 15) and PGE_3_ (Eq. (16)) utilize the enzyme $e_{Ptges}$. PGH_2_ gets converted into PGD_2_ and PGE_2_ (Equation 11), and PGH_3_ gets converted into PGD_3_ and PGE_3_ (Equation 12). The third and fourth terms in Equation 11 denote the PGD_2_ and PGE_2_ production rate from the substrate PGH_2_, respectively, where $k_{PGD_{2}}$ and $k_{PGE_{2}}$ are the rate constants. Likewise, the third and fourth terms in Equation 12 are the formation rates of PGD_3_ and PGE_3_ from PGH_3_, where $k_{PGD_{3}}$ and $k_{PGE_{3}}$ are the corresponding rate constants. $e_{COX},e_{Ptgds}$, and $e_{Ptges}$ indicate the dynamic enzyme levels. Their rate balance equations are shown later in supplementary section 1. There are no cybernetic variables associated with reactions (Equations (13), (14), (15), (16)).

(13) $$PGH_{2}+e_{Ptgds}\overset{k_{PGD_{2}}}{\to }\;PGD_{2}$$

(14) $$PGH_{3}+e_{Ptgds}\overset{k_{PGD_{3}}}{\to }\;PGD_{3}$$

(15) $$PGH_{2}+e_{Ptges}\overset{k_{PGE_{2}}}{\to }\;PGE_{2}$$

(16) $$PGH_{3}+e_{Ptges}\overset{k_{PGE_{3}}}{\to }\;PGE_{3}$$

Consequently, the fluxes downstream (third and fourth terms) of PGH_2_ arise from the synthesis of PGD_2_ and PGE_2_, while for PGH_3_, they are a result of PGD_3_ and PGE_3_ formation.

### Enzyme balance in the cybernetic modeling framework

The rate balance for the enzyme, $e_{COX}$, shared by PGH_2_ and PGH_3_, is given by Equation 17.

(17) $$\frac{d[e_{COX]}}{dt}=\alpha +u_{PGH_{2}}\frac{k_{ePGH_{2}}\left[AA\right]}{Km_{AA}+\left[AA\right]}+u_{PGH_{3}}\frac{k_{ePGH_{3}}\left[EPA\right]}{Km_{EPA}+\left[EPA\right]}-\beta \left[e_{COX}\right]$$

α denotes the constitutive rate of formation of COX1. The second and third terms denote the inducible rate of synthesis of COX2. The cybernetic formulation assumes that enzymes depend on the levels of the substrates they react with. Hence, the inducible rate (second and third terms) for $e_{COX}$ is a function of AA and EPA. It follows Michaelis-Menten kinetics and accounts for the enzyme synthesis control for products PGH_2_ and PGH_3_ by the variables $u_{PGH_{2}}$ and $u_{PGH_{3}}$, respectively (27). $k_{ePGH_{2}}$ and $k_{ePGH_{3}}$ are the maximum rates for Michaelis-Menten kinetics, modulated by $u_{PGH_{2}}$ and $u_{PGH_{3}}$, respectively. β is the degradation rate of the enzymes. $Km_{AA}$ and $Km_{EPA}$ are Michaelis-Menten constants for AA and EPA, respectively. The enzyme balance for $e_{Ptgds}$, $e_{Ptges}$, and $e_{dhkPGD_{2}}$ are included in supplemental section 1.

### Simulation strategy

The model comprised a system of 13 ordinary differential equations (ODEs) and involved 41 parameters. To estimate these parameters, a two-step hybrid optimization approach was used. The initial parameter estimation used the pattern search method (implemented using the MATLAB® function "patternsearch"), followed by further parameter optimization using the fmincon function ("fmincon" in MATLAB®). Due to the instability of intermediates PGH_2_ and PGH_3_, no measurements were available for these metabolites. Therefore, their concentrations were assumed to be less than 10 pmol/μg DNA, and their profiles were constrained accordingly. Additionally, the initial conditions for these intermediates were forced to be the same for both experimental conditions, as all other metabolites had similar starting levels at zero minutes. The experimental data consisted of 8 time points spanning a duration of 60 min. These data points were used to determine the model parameters. The scaled fit-error between the experimental data ($y_{i,j,\exp}$) and the simulated data ($y_{i,j,pred}$) was minimized using Equation 18, which served as the cost function.

(18) $$\underset{K,X_{o}}{\mathrm{Min}}\left(\sum_{i=1}^{nsp}\left(\sum_{j=1}^{ni}\frac{{\left(y_{i,j,\exp}-y_{i,j,pred}\left(K,X_{0}\right)\right)}^{2}}{\max\left(y_{i,j,\exp}\right)}\right)\right)$$

where K represents the parameters, $X_{0}$ denotes the initial condition of enzyme concentrations, ni is the number of time points, 8 (indexed as *j*), and nsp is the total number of species (indexed as i). The ode15s was used to solve the ODEs in MATLAB (2019, Natick, MA). The parameters were optimized, and the EPA-supplemented scenarios for all metabolites were simulated using the estimated parameters.

### Model validation

#### F-test

We performed F-test, using Equation 19, to assess the goodness of fits of simulated profiles to the experimental observations for AA and EPA-supplemented cases (20).

(19) F=(∑j=1nt(Yjtrt−X¯jtrt)2+∑j=1nt(Yjctrl−X¯jctrl)2)(ne×nt)(∑j=1nt∑i=1nr(Xijtrt−X¯jtrt)2+∑j=1nt∑i=1nr(Xijctrl−X¯jctrl)2)(ne×nt×(nr−1))

where $X_{j}$, ${\bar{X}}_{j}$, and $Y_{j}$ denote the experimental data, mean experimental data, and simulated (fitted) data at time point *j*, respectively. nr is the number of replicates (nr=3, indexed as i), nt is the number of time points (nt=7, index j). ne is the number of experimental conditions used, and trt and ctrl are treatment (EPA supplemented) and control groups, respectively (ne=2). The degrees of freedom for determining the F distribution are df_1_ = (ne×nt) and df_2_ = (ne×nt×(nr−1)).

#### Leave-one-out-metabolite PGD_2_ cross-validation

The leave-one-out metabolite cross-validation method is used to assess the performance of the cybernetic model developed. In this case, a prediction is made for a metabolite not used to train the model. For instance, the metabolite PGD_2_ is removed from the objective function, Equation 18, and the profiles for other metabolites are predicted. The goodness of fit of the predicted PGD_2_ profile to the experimental data determines the model performance (21).

#### Prediction for an unseen case of DHA supplementations

The cybernetic model developed for EPA-supplemented situation can be modified to study the previously unobserved case of DHA-supplemented scenarios. The AA and DHA metabolism network include the AA branch (as shown in Fig. 1) and a downstream product of DHA, $P_{D}$, an EFOX (62). The measurements are unavailable for downstream DHA products (59). We assumed that $P_{D}$ has no further conversions. Following the AA and EPA case, the proposed cybernetic objective for AA and DHA metabolic network is to maximize the combined rates of AA and DHA consumption. This cybernetic goal is equivalent to the optimization of the combined production rates of PGH_2_ and $P_{D}$ (Equations 20 and 21). We depict the outflux (consumption rate) of AA and DHA by $\rho_{AA}^{out}$ and $\rho_{DHA}^{out}$, respectively (Equation 20). Similarly, the influx (production rate) of PGH_2_ is denoted by $\rho_{PGH_{2}}^{in}$, and $P_{D}$ by $\rho_{P_{D}}^{in}$ (Equation 20). The definitions of $\rho_{PGH_{2}}^{in}$ and $\rho_{P_{D}}^{in}$ are shown in Equation 21. The cybernetic goal is (Equation 20):

(20) $$\max\;\left[\rho_{AA}^{out}+\rho_{DHA}^{out}\right]=\max\;\left[\rho_{{PGH}_{2}}^{in}+\rho_{P_{D}}^{in}\;\right]$$

where

(21) $$\rho_{PGH_{2}}^{in}=k_{PGH_{2}}\left[e_{COX}\right]\left[AA\right];\;\rho_{P_{D}}^{in}=k_{P_{D}}\left[e_{COX}\right]\left[DHA\right]$$

The modified control variable definitions for PGH_2_ ($v_{PGH_{2}}$, $u_{PGH_{2}}$) and $P_{D}$ ($v_{P_{D}}$, $u_{P_{D}}$) are shown in Equations 22 and 23, respectively.

(22) $$v_{PGH_{2}}=\frac{\rho_{PGH_{2}}^{in}}{\max\left(\rho_{PGH_{2}}^{in},\rho_{P_{D}}^{in}\right)};\;v_{P_{D}}=\frac{\rho_{P_{D}}^{in}}{\max\left(\rho_{PGH_{2}}^{in},\rho_{P_{D}}^{in}\right)}$$

(23) $$u_{PGH_{2}}=\frac{\rho_{PGH_{2}}^{in}}{\rho_{PGH_{2}}^{in}+\rho_{P_{D}}^{in}};\;u_{P_{D}}=\frac{\rho_{P_{D}}}{\rho_{PGH_{2}}^{in}+\rho_{P_{D}}^{in}}$$

The rate balance for all the metabolites of the AA branch, PGH_2_, PGD_2_, PGE_2_, dhkPGD_2_, PGJ_2_, and dPGJ_2_, are in accordance with the kinetic balance equations observed in the EPA-supplemented scenario (included in supplemental section 1). Similar to the EPA-supplemented case, a cybernetic variable is associated with the enzyme forming $P_{D}$ from substrate DHA (Equation 24). The downstream fluxes of $P_{D}$ are zero.

(24) $$\frac{d\left[P_{D}\right]}{dt}=k_{P_{D}}v_{P_{D}}\left[DHA\right]\left[e_{COX}\right]\left(1+k_{ATP}\left[ATP\right]\right)-g_{P_{D}}\left[P_{D}\right]$$

where $k_{P_{D}}$ is the rate constant of the reaction, $v_{P_{D}}$ is the cybernetic variable for modulating enzyme activity, and $g_{P_{D}}$ is the decay rate of $P_{D}$. Due to the absence of subsequent conversions of product $P_{D}$, the modified enzyme rate balances for AA and DHA scenario are Equation 25. Readers are referred to supplemental section 3 for the full set of equations. Equation 25 demonstrates the enzyme balance for $e_{COX}$. The inducible rate is due to the substrates AA and DHA. $k_{eP_{D}}$ is the maximum inducible rate and $Km_{DHA}$ is the Michaelis-Menten constant for DHA. The other constants hold the same meaning mentioned previously. We simulated the system using the above-mentioned simulation strategy and determined the parameters.

(25) $$\frac{d\left[e_{COX}\right]}{dt}=\alpha +u_{PGH_{2}}\frac{k_{ePGH_{2}}\left[AA\right]}{Km_{AA}+\left[AA\right]}+u_{P}\frac{k_{eP_{D}}\left[DHA\right]}{Km_{DHA}+\left[DHA\right]}-\beta \left[e_{COX}\right]$$

## Results

### Simulation results

**Fig. 3:**
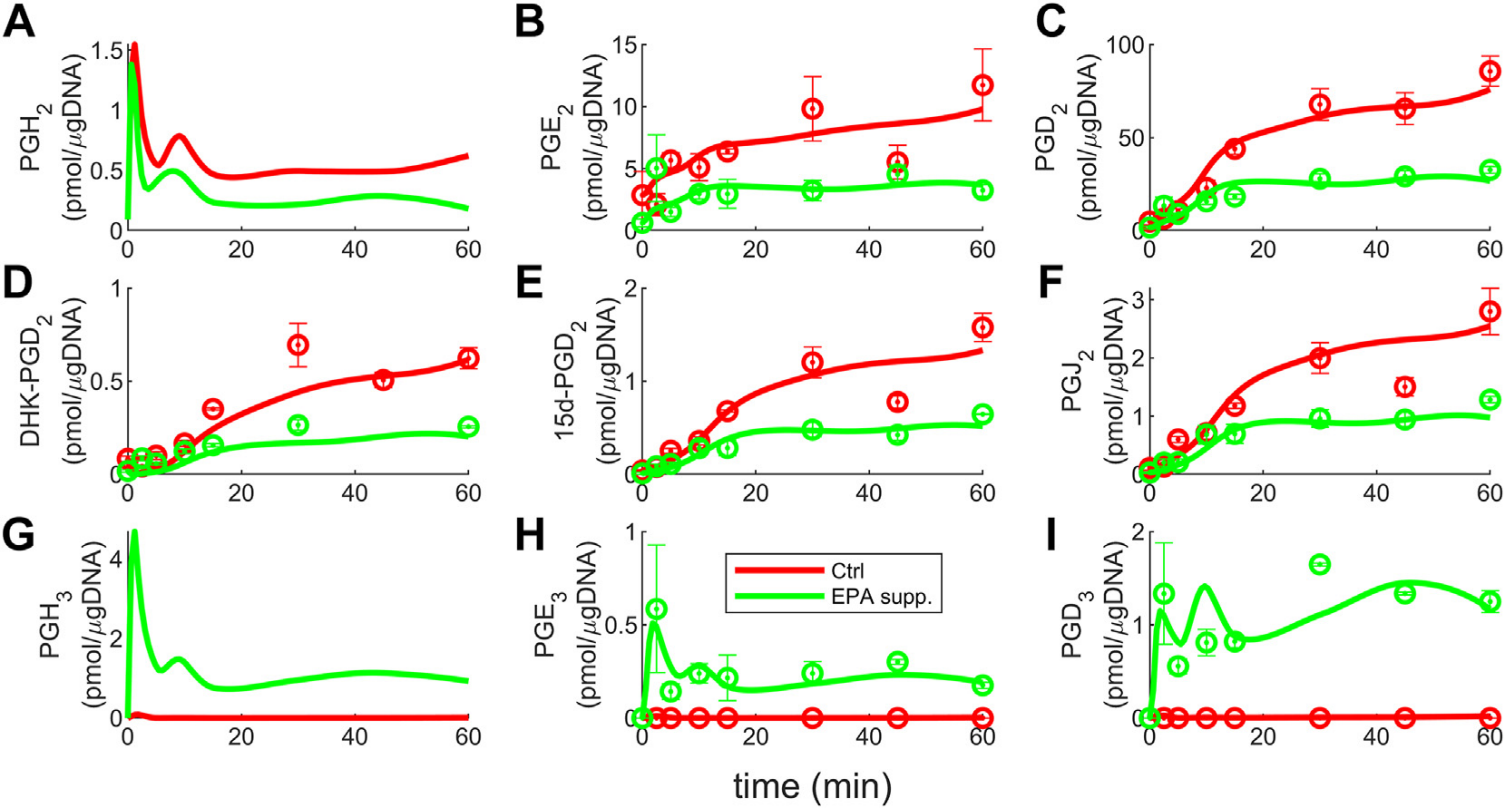
The cybernetic model simulation results obtained for EPA addition experimental conditions. The model successfully captured the nonlinear trends exhibited by all the metabolites. PGH_2_ (A) and PGH_3_ (G) measurements are unavailable due to their unstable existence. Their levels are constrained to remain below 10 pmol/μg DNA. With EPA supplementation, there is a decrease in the levels of 2-series PGs (B–F) and an increase in the levels of 3-series PGs (H–I). The change occurs because EPA and PGH_3_ become available to contribute to the production of 3-series PGs. This observation highlights the competition for the shared enzyme $e_{COX}$ that reacts with different substrates, AA and EPA. Similarly, PGH_2_ (A) decreases upon EPA addition, while PGH_3_ (G) increases, further illustrating the effect of the competition between these substrates for the enzyme $e_{COX}$. AA, arachidonic acid; EPA, eicosapentaenoic acid; PG, prostaglandin.

The cybernetic model captures and explains the competition between enzymes in the metabolism of AA and EPA, leading to the formation of 2-series and 3-series PGs, respectively. The estimated parameters are included in supplemental section 2. The enzyme COX ($e_{COX}$) is involved in both AA and EPA metabolism, while $e_{Ptgds}$ is associated with PGD_2_ and PGD_3_, and $e_{Ptges}$ is linked to PGE_2_ and PGE_3_ (21, 53, 59). To simplify the model, we focus on the initial level of enzyme competition, where COX ($e_{COX}$) is shared by AA and EPA. Consequently, we define cybernetic variables $\left(u_{PGH_{2}},v_{PGH_{2}}\right)$ and $\left(u_{PGH_{3}},v_{PGH_{3}}\right)$ for PGH_2_ and PGH_3_, respectively. Experimental data are available for cases where the cells are supplemented with EPA and DHA. The cybernetic model is trained using EPA supplementation data and predictions are made for the DHA supplementation scenario. The results for EPA supplementation are presented in Fig. 3, which demonstrates a good fit between the model prediction and experimental data. However, measurements for PGH_2_ and PGH_3_ are not available due to their inherent instability. Hence, the simulated profiles of PGH_2_ and PGH_3_ in Fig. 3A, G are constrained to be less than 10 pmol/μg DNA. The plots reveal higher levels of 2-series PGs in the control (Ctrl) scenario (red curve, Fig. 3B–F), and the presence of EPA in the EPA-supplemented case reduces the synthesis of 2-series PGs (green curve, Fig. 3B–F). In contrast, negligible levels of 3-series PGs are observed in the control scenario (red curve, Fig. 3 3H, I), while their concentrations increase with EPA supplementation (green curve, Fig. 3H, I). These findings are consistent with the competitive nature of AA and EPA for the same enzyme COX shown in Figs. 1 and 2. Similarly, the profiles of PGH_2_ (Fig. 3A) and PGH_3_ (Fig. 3G) are also consistent with the enzyme competition, with PGH_2_ decreasing upon EPA addition while PGH_3_ increases. Upon supplementation of EPA, the levels of AA and EPA become similar, and the reaction flux (represented as ρ) resulting from EPA metabolism to form PGH_3_ is higher than that of AA producing PGH_2_. This difference can be attributed to the higher rate constant of PGH_3_ formation ($k_{PGH_{3}}$) compared to the rate constant for PGH_2_ formation ($k_{PGH_{2}}$). The elevated flux of PGH_3_ indicates that the enzyme COX ($e_{COX}$) exhibits a greater enzyme activity for EPA than AA when EPA is supplemented (21). This competitive behavior sheds light on the mechanistic relationship between the enzymes and substrates involved. The dynamics of the cybernetic variables governing enzyme activity and synthesis support this observation. These dynamics will be elaborated upon in the subsequent section, providing further insight into the implications of enzyme activity plots.

The nonlinear trends of the 2-series PGs (Fig. 3B–F) and 3-series PGs (Fig. 3H, I) are accurately captured by our cybernetic model, as indicated by the F-test values (discussed later). The nonlinearity in the kinetics of PGH_2_ and PGH_3_ arises from the influence of the cybernetic control variables. The profiles of AA and EPA, which serve as inputs to our model, can be found in the supplementary material (supplemental Figs. S1 and S2). In both experimental scenarios, the AA profile follows similar trends, resulting in comparable behaviors of the red and green curves for the 2-series PGs. The AA levels increase significantly until 30 min, after which they plateau, leading to similar downstream behavior of the 2-series PGs. EPA is initially introduced at a relatively high concentration, resulting in significant production of 3-series PGs at 2.5 min. However, as time progresses, the decline in EPA levels also leads to a decrease in 3-series PGs. Similarly, the nonlinear trend observed in the 3-series PGs at later time points follows the EPA profile.

### Dynamics of cybernetic control variables

Our cybernetic model accounts for the unknown regulatory steps by introducing the control variables for PGH_2_ and PGH_3_ formation reactions. The enzyme $e_{COX}$ is responsible for catalyzing the conversion of AA and EPA into PGH_2_ and PGH_3_, respectively. Since AA and EPA may have different enzyme synthesis regulation ($u_{PGH_{2}}$, $u_{PGH_{3}}$) and enzyme activity modulation ($v_{PGH_{2}}$, $v_{PGH_{3}}$), they are assigned distinct control variables. Following the matching and proportion laws, $u_{i}$ and $v_{i}$ are directly proportional to flux ($\rho_{i}$), and vary by their respective normalizations. The plots for $u_{PGH_{2}}\left(u_{PGH_{3}}\right)$ and $v_{PGH_{2}}\left(v_{PGH_{3}}\right)$ show a similar trend, and are consistent with the definitions of *u* and *v*.

**Fig. 4:**
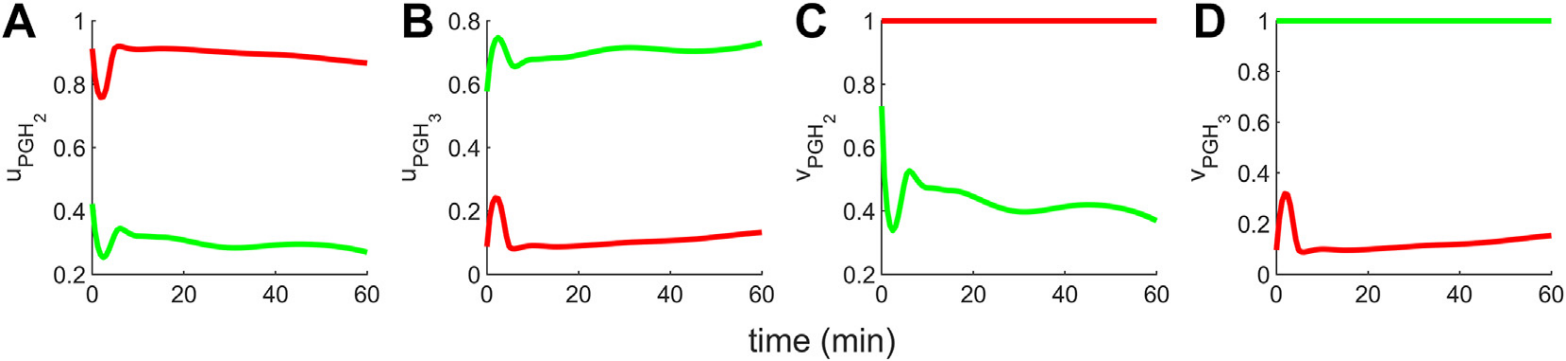
The dynamics of cybernetic control variables for PGH_2_ ($u_{PGH_{2}}$, $v_{PGH_{2}}$) and PGH_3_ ($u_{PGH_{3}}$, $v_{PGH_{3}}$). PGH_2_ is the dominant metabolite for the Ctrl case because $u_{PGH_{2}}>\;u_{PGH_{3}}$ (A and B red curve) and $v_{PGH_{2}}>v_{PGH_{3}}$ (C and D red curve). However, upon EPA supplementation, PGH_3_ is the leading product because $u_{PGH_{3}}>\;u_{PGH_{2}}$ (A and B green curve) and $v_{PGH_{3}}>v_{PGH_{2}}$ (C and D green curve). The switch from PGH_2_ to PGH_3_ dominance captures the COX enzyme competition. The synthesis and activity of COX prioritize an increased catalytic effect on EPA relative to AA when there is EPA supplementation. AA, arachidonic acid; COX, cyclooxygenase; EPA, eicosapentaenoic acid; PG, prostaglandin.

The metabolite with the highest flux is defined as dominant. In the control case (Ctrl) where AA is present at basal levels and EPA is negligible, PGH_2_ emerges as the dominant metabolite. This is evident from the comparison $u_{PGH_{2}}>\;u_{PGH_{3}}$ (Fig. 4A, B red curve) and $v_{PGH_{2}}>v_{PGH_{3}}$ (Fig. 4C, D red curve). Such dominance of PGH_2_ is expected in the context of Ctrl, where EPA levels are insignificant. Conversely, with EPA supplementation, PGH_3_ becomes the leading metabolite, as indicated by $u_{PGH_{3}}>\;u_{PGH_{2}}$ (Fig. 4A, B green curve) and $v_{PGH_{3}}>v_{PGH_{2}}$ (Fig. 4C, D green curve). This behavior supports the proposition that COX ($e_{COX}$) reacts with EPA more potently than AA when the system is supplemented with EPA, owing to enhanced enzyme synthesis and activity control. It is important to note that we observed a switch from dominant proinflammatory PGH_2_ action for control to antiinflammatory PGH_3_ for the EPA-supplemented case. The reason for the switch is discussed in the next section.

### Switching between the dominance of the cybernetic control variables $v_{\mathrm{PG}H_{2}}$ and $v_{\mathrm{PG}H_{3}}$

**Fig. 5:**
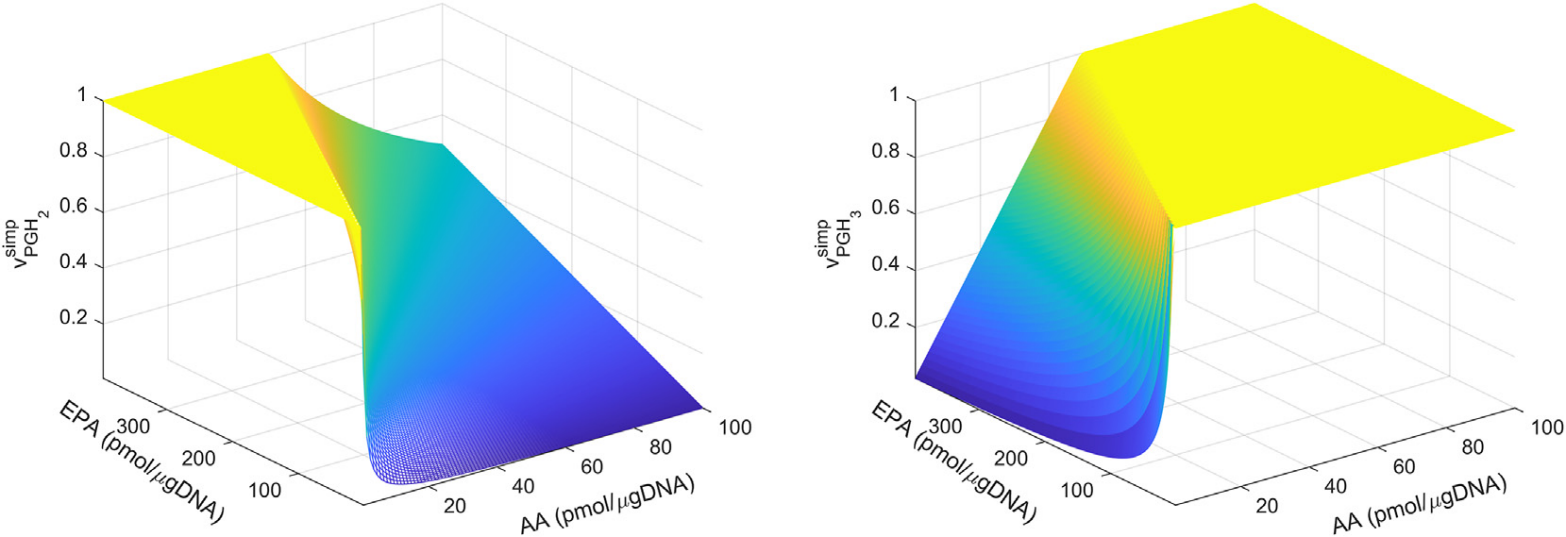
The surface plot depicts the relationship between simplified control variables for enzyme activity ($v_{PGH_{2}}^{simp}$ , $v_{PGH_{3}}^{simp}$), and AA and EPA concentrations. $v_{PGH_{2}}^{simp}$ dominates for higher AA and $v_{PGH_{3}}^{simp}$ takes precedence for higher EPA levels. Consequently, the system switches from $v_{PGH_{2}}^{simp}=1$ to $v_{PGH_{3}}^{simp}=1$ as EPA increases. The ratio of switch levels, $[A{A]}_{swtich}$ to ${\left[EPA\right]}_{switch}$, follows (Equation 27). Therefore, AA and EPA levels are responsible for the transition in dominance of proinflammatory PGH_2_ in the Ctrl scenario to antiinflammatory PGH_3_ in the EPA-supplemented case (Fig. 4). AA, arachidonic acid; EPA, eicosapentaenoic acid; PG, prostaglandin.

The control variables for activity, $v_{PGH_{2}}$ and $v_{PGH_{3}}$ (Equations 3 and 5), depend on the AA, EPA, and $e_{COX}$ levels. As $e_{COX}$ is utilized by both AA and EPA, due to the ratio involved, $v_{PGH_{2}}$ and $v_{PGH_{3}}$ become independent of the $e_{COX}$ concentration; hence, $v_{PGH_{2}}$ and $v_{PGH_{3}}$ can be analytically simplified as $v_{PGH_{2}}^{simp}$ and $v_{PGH_{3}}^{simp}$, respectively. It is important to note that $v_{PGH_{2}}^{simp}=v_{PGH_{2}}$ and $v_{PGH_{3}}^{simp}=v_{PGH_{3}}$. The simulation results from Section 3.1 provide $k_{PGH_{2}}$ and $k_{PGH_{3}}$, which are utilized in this analysis. Figure 5 shows the variation of $v_{PGH_{3}}^{simp}$ and $v_{PGH_{2}}^{simp}$ (Equation 26) with AA and EPA concentrations. As expected, $v_{PGH_{2}}^{simp}$ reaches a maximum value of 1 for higher concentrations of AA, whereas $v_{PGH_{3}}^{simp}$ is maximized for higher EPA levels. Equation 27 analytically describes the ratio of ${\left[EPA\right]}_{switch}$ to $[A{A]}_{swtich}$, which causes the transition from the dominance of $v_{PGH_{3}}^{simp}$ to $v_{PGH_{2}}^{simp}$. The ratio is 2.65 in this study, indicating $v_{PGH_{3}}^{simp}$ prevails over a broader range of AA and EPA levels (Fig. 5).

(26) $$v_{PGH_{2}}^{simp}=\frac{k_{PGH_{2}}\left[AA\right]}{\max\left(k_{PGH_{2}}\left[AA\right],{\;k}_{PGH_{3}}\left[EPA\right]\right)}\;;v_{PGH_{3}}^{simp}=\frac{k_{PGH_{3}}\left[EPA\right]}{\max\left(k_{PGH_{2}}\left[AA\right],{\;k}_{PGH_{3}}\left[EPA\right]\right)}$$

(27) $$[A{A]}_{swtich}k_{PGH_{2}}={\left[EPA\right]}_{switch}k_{PGH_{3}};\;\frac{[A{A]}_{swtich}}{[EP{A]}_{switch}}=\frac{k_{PGH_{3}}}{k_{PGH_{2}}}$$

### Cybernetic model prediction: enzyme dynamics

**Fig. 6:**
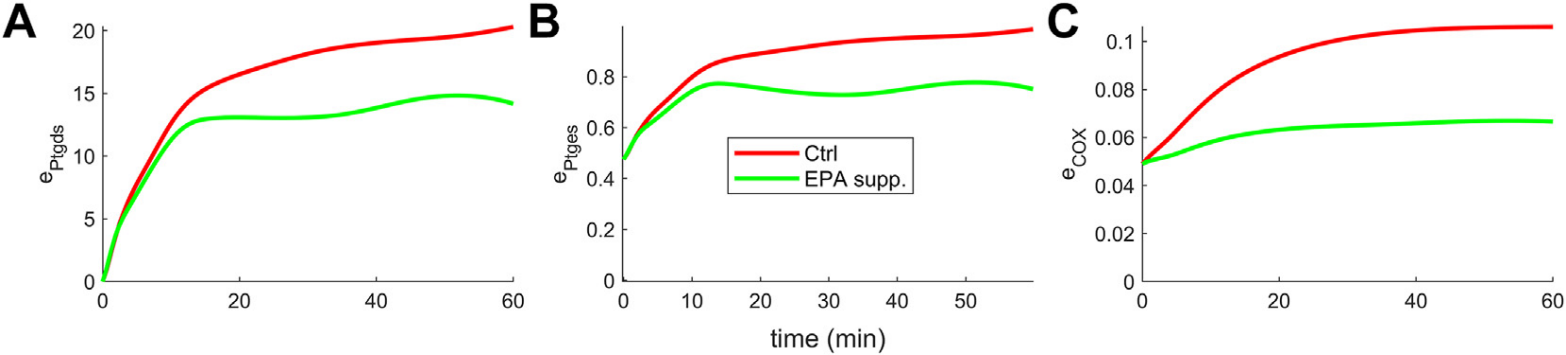
The cybernetic model provides predictions for the enzyme profiles. Among these enzymes, $e_{Ptgds}$ (A) stands out with higher levels compared to the others. Its levels increase and reach saturation following the profiles of PGD_2_ (Fig. 3C) and PGD_3_ (Fig. 3I), respectively. Similarly, the enzymes $e_{Ptges}$ (B) and $e_{COX}$ (C) show an increase in levels due to the corresponding trends observed in their substrates PGE_2_, PGE_3_, and AA, EPA, respectively. These patterns hold for both experimental conditions of Ctrl and EPA supplementation. AA, arachidonic acid; EPA, eicosapentaenoic acid; PG, prostaglandin.

A notable aspect of the cybernetic model is that it can predict enzyme profiles. The enzyme $e_{Ptgds}$, which is depicted in Fig. 6A, is responsible for catalyzing the production of PGD_2_ and PGD_3_. In comparison to other enzymes, $e_{Ptgds}$ shows higher levels. This trend is primarily because the levels of PGD_2_ and PGD_3_ are higher among the 2-series (as shown in Fig. 3C) and 3-series PGs (as shown in Fig. 3I), respectively. $e_{Ptgds}$ increases and eventually saturates, reflecting the similar trends observed for prominent PGD_2_ and PGD_3_. Similarly, $e_{Ptges}$ (Fig. 6B) also increases because of rising PGE_2_ and PGE_3_ levels. The enzyme $e_{COX}$ (Fig. 6C) also follows a similar pattern, which depends on AA and EPA levels.

### Model validation results

#### F-test

The F-test for all metabolites show that the fit error is less than the experimental error as all F-test values are less than F_0.05_ (16, 32) = 0.46. The 2-series metabolites, including PGD_2_, PGJ_2_, and dPGD_2_, exhibit a better fit compared to PGE_2_ and dhkPGD_2_, and all 3-series PGs demonstrate a satisfactory fit.

#### Leave-one-out-metabolite PGD_2_ cross-validation method

**Fig. 7:**
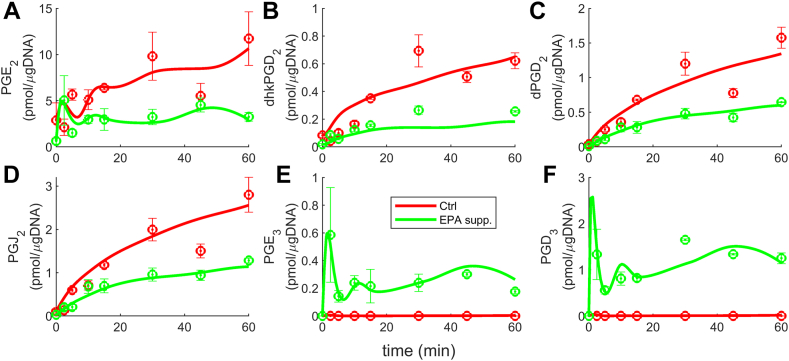
The simulation results for the model with the leave-one-out-metabolite PGD_2_ cross-validation method. The plots (A–F) resemble the original AA and EPA addition instances (Figs. 3A–F and 7A–C). Despite removing PGD_2_ (Equation 13) from the cost function, the results remain unaffected due to the adherence of all metabolites to the kinetic rate balance. The similarity of results to the original ones implies that our cybernetic model suitably incorporated mechanistic details. AA, arachidonic acid; EPA, eicosapentaenoic acid; PG, prostaglandin.

The model demonstrates a good fit to the data when using the leave-one-out-metabolite PGD_2_ cross-validation method. (Fig. 7A–F). The results align closely with the original AA and EPA-supplemented conditions, validating the cybernetic formulation. Removing PGD_2_ from the cost function (Equation 13) does not significantly degrade the quality of the fit results as PGD_2_ is included in the reaction kinetics and satisfies flux balances for all metabolites. The goodness of fits indicates that our cybernetic model accurately captured the mechanistic details, even without considering PGD_2_ in the cost function. The rationales for the trends observed remain the same for metabolites and enzymes (Fig. 7A–F).

#### Validation of the model by the application of the cybernetic model developed to a new scenario: DHA supplementation

**Fig. 8:**
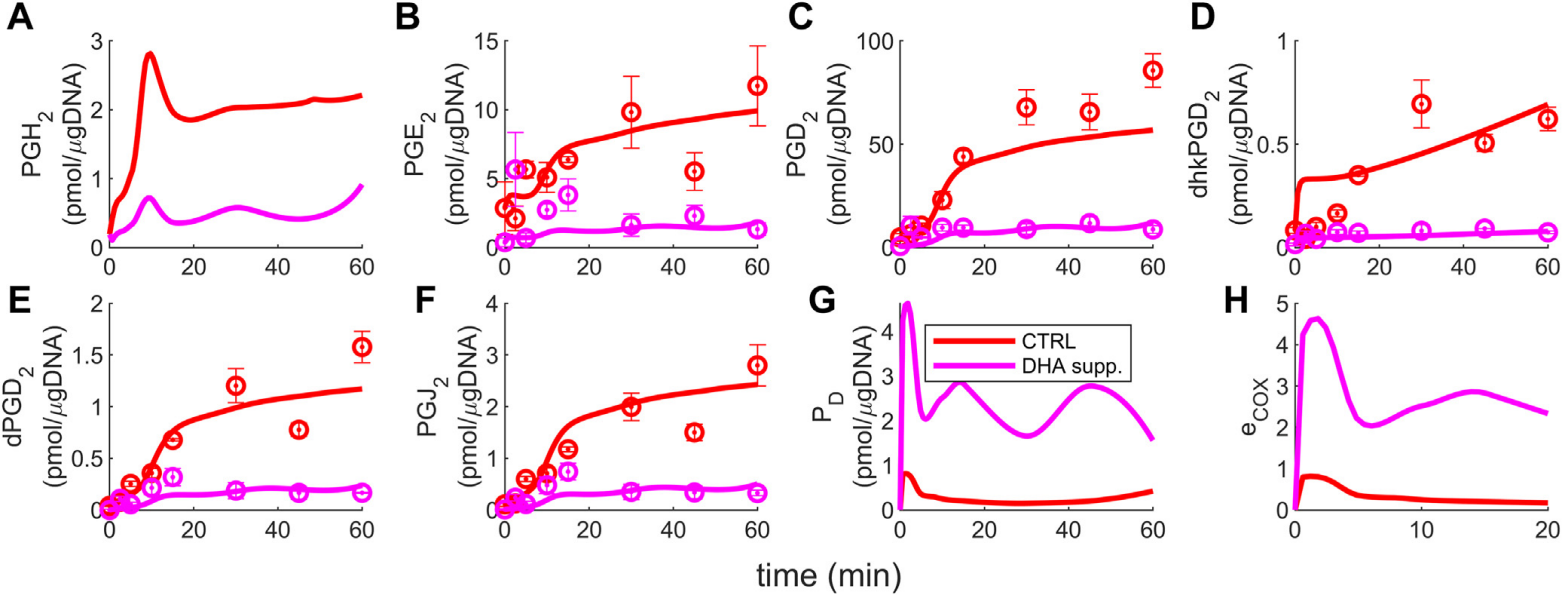
The AA and DHA supplementation case simulation results demonstrate similar trends to the AA and EPA addition scenarios. Specifically, there is a decrease in the levels of 2-series PGs (B–F) due to increased DHA levels. The competition between PGH_2_ and PGH_3_ is maintained, as evidenced by the decline in PGH_2_ (A) and the increase in P_D_ (G) in the DHA addition condition. The $e_{COX}$ levels are shown in the subplot (H). AA, arachidonic acid; DHA, docosahexaenoic acid; EPA, eicosapentaenoic acid; PG, prostaglandin.

**Fig. 9:**
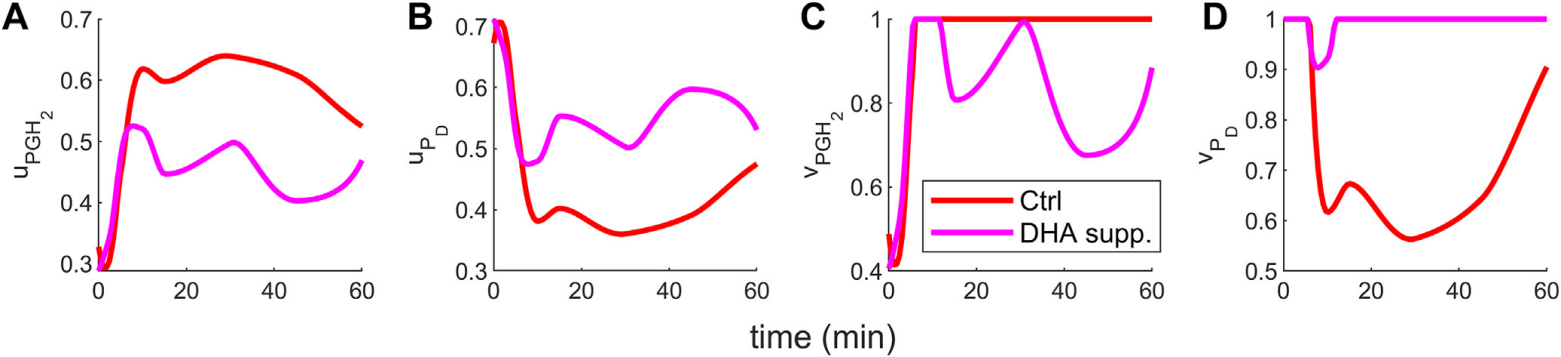
The cybernetic control variables for PGH_2_ and $P_{D}$ for control condition and with DHA supplementation. In the control condition, PGH_2_ emerges as the primary metabolite, characterized by higher levels of $u_{PGH_{2}}$ (A) compared to $u_{P_{D}}$ (B), and higher levels of $v_{PGH_{2}}$ (C) compared to $v_{P_{D}}$(D). This indicates a prevailing proinflammatory action in this condition. In contrast, in the DHA-supplemented scenario, similar to the case of EPA supplementation, the downstream metabolite of DHA, $P_{D}$, becomes the dominant metabolite. This is evident from higher levels of $u_{P_{D}}$ (B) compared to $u_{PGH_{2}}$, (A), and higher levels of $v_{P_{D}}$ (D) compared to $v_{PGH_{2}}\text{.}$ (C) Consequently, an antiinflammatory role is prevalent in this scenario. DHA, docosahexaenoic acid; EPA, eicosapentaenoic acid; PG, prostaglandin.

We simulated the control and DHA-supplemented model, presented in the Materials and methods section. As discussed, measurements are unavailable for metabolites formed due to DHA metabolism, so we assumed that $P_{D}$ (an EFOX) is the only downstream product. The results are shown in Fig. 8. The fits are reasonable, increasing our confidence in the cybernetic model. Similar to EPA, DHA is also antiinflammatory. In these supplementations, we observed a decline in 2-series PGs levels due to DHA addition (pink curve, Fig. 8B–F). PGH_2_ and $P_{D}$ competition is also intact: PGH_2_ decreases (Fig. 8A), and $P_{D}$ increases (Fig. 8G) with DHA addition. The reasoning behind the dynamics of metabolites and enzymes aligns with the AA and EPA supplementation, except for downstream $P_{D}$ due to the absence of its downstream metabolites. For AA and DHA experiments, $u_{PGH_{2}}>\;u_{P_{D}}$ (Fig. 9A) and $v_{PGH_{2}}>v_{P_{D}}$ (Fig. 9C) indicates the prominence of PGH_2_ in the control condition. Whereas, for DHA supplementation case (Fig. 9B, D), $P_{D}$ is the dominant metabolite, promoting the antiinflammatory behavior. The observed result is consistent with the EPA supplementation scenario, where a similar pattern was observed. In the control condition, PGH_2_ emerged as the prominent metabolite, whereas in the EPA supplemented scenario, PGH_3_ dominated.

## Discussion

This study presents a mathematical model that aims to capture the dynamics of enzyme competition in the metabolism of AA and EPA/DHA. We used the cybernetic model, which inherently incorporates the enzyme competition mechanism by optimizing the cybernetic goal. Within this framework, AA and EPA share the enzyme COX ($e_{COX}$). AA and its downstream 2-series PGs demonstrate proinflammatory characteristics, while EPA and the 3-series PGs are associated with antiinflammatory behavior (35, 53). A recent study by Zaid *et al.* observed high levels of both series PGs in intubated COVID-19 patients (60). This finding emphasizes the critical roles of both proinflammatory and antiinflammatory stages in the inflammatory response observed in these individuals. Considering the important opposing functionality of AA and EPA, we hypothesized that during the inflammatory response in RAW 264.7 cells, the biological objective is to maximize both the proinflammatory and antiinflammatory phases. Consequently, we proposed the cybernetic goal: maximizing the combined consumption rates of AA (representing proinflammatory action) and EPA (representing antiinflammatory activity). This goal formulation is equivalent to maximizing the production rates of PGH_2_ and PGH_3_ since they are the primary products of AA and EPA through $e_{COX}$ activation, respectively. The model fitted the experimental data reasonably, supporting our hypothesis regarding the biological goal and cybernetic goal formulation. The success of the model underscores the goal-oriented behavior of the system, where the levels and activities of relevant enzymes are modulated to achieve the formulated goal (27). The quantitative results of this study emphasize the significance of both pro inflammatory and antiinflammatory phases during the inflammatory response in RAW 264.7 macrophages.

The cybernetic control variables are introduced for the enzyme COX ($e_{COX})$, involved in forming PGH_2_ and PGH_3_ from AA and EPA, respectively. It should be noted that the cybernetic variables are the ratios of fluxes and do not represent fluxes themselves. They are lumped variables that capture the complex biological regulation mathematically. The single enzyme $e_{COX}$ concerned with the production of PGH_2_ and PGH_3_ is assigned different variables for enzyme activity ($v_{PGH_{2}}$, $v_{PGH_{3}}$) and synthesis ($u_{PGH_{2}}$, $u_{PGH_{3}}$). This formulation accounts for the potentially distinct processes governing the activity and synthesis process with which $e_{COX}$ interacts with AA and EPA. Two experimental conditions of control and EPA supplementation are explored. The enzyme competition is depicted by the dynamics of the cybernetic control variables for enzyme activity ($v_{PGH_{2}}$, $v_{PGH_{3}}$), where upon EPA supplementation, the activity of PGH_3_ increases and is higher than PGH_2_. The simplified $v_{PGH_{2}}^{sim}$ and $v_{PGH_{3}}^{sim}$ also demonstrate similar trend. Consequently, the addition of EPA shifts the system from the dominance of the proinflammatory 2-series PGs to antiinflammatory 3-series PGs. The surface plot of simplified control variables ($v_{PGH_{2}}^{sim}$, $v_{PGH_{3}}^{sim}$) further illustrates that the levels of AA and EPA play a vital role in the shift from the dominance of proinflammatory PGH_2_ in the Ctrl scenario to the prevalence of antiinflammatory PGH_3_ in the EPA-supplemented case. The ratio of levels of ${\left[AA\right]}_{switch}$ to ${\left[EPA\right]}_{switch}$ where switch occurs is 3.75, indicating that $v_{PGH_{3}}^{simp}$ starts to prevail for smaller EPA levels; hence, $v_{PGH_{3}}^{simp}$ is dominant over a wider range of AA and EPA levels. The cybernetic model adequately described the experimental data, demonstrating that incorporating regulatory variables for $e_{COX}$ is sufficient for studying AA and EPA metabolism. Moreover, it illustrates that an appropriate control mechanism for $e_{COX}$ effectively explains the subsequent conversion of PGH_2_ and PGH_3_ to downstream 2-series and 3-series PGs, respectively. The outcome of the model, with an ${\left[AA\right]}_{switch}$ to ${\left[EPA\right]}_{switch}$ ratio of 2.65, falls within the desirable range for promoting an antiinflammatory response and thereby reducing the risk of diseases. Recent studies have shown that populations in regions with an AA to EPA fatty acid ratio ranging from 1 to 5 experience fewer chronic diseases compared to those in areas where n-6 fatty acids dominate, such as in Western diets with ratios as high as 17 (53, 63).

The cybernetic model formulated for enzyme competition fitted the experimental data well. Our model successfully fitted the data points that were not captured by the Michaelis-Menten dynamics, compared to the previous study by Gupta *et al.* (21) based on Michaelis-Menten kinetics. For instance, the model predictions were far off the initial peak observed for PGD_3_ and PGE_3_ in the case of EPA supplementation. In contrast, our model successfully fitted these data points, as supported by the F-test values. The improved performance can be attributed to the incorporation of known and unknown regulatory processes through cybernetic control variables. Notably, the cybernetic model also offered the added advantage of including and predicting enzyme dynamics, overcoming the limitation of unavailable enzyme measurements. The model formulation included the kinetic rate balances for enzymes $e_{Ptgds}$, $e_{Ptges}$, and $e_{COX}$, and their dynamics are shown in Fig. 6: $e_{Ptgds}$ (Fig. 6A), $e_{Ptges}$ (Fig. 6B), and $e_{COX}$ (Fig. 6C). The rationale behind the model effectively capturing the initial peak of PGH_2_/PGH_3_, PGD_2_/PGD_3_, and PGE_2_/PGE_3_ is as follows. For Ctrl, PGH_2_ increased for t<2.5 minutes because the influx of PGH_2_ ($v_{PGH_{2}}k_{PGH_{2}}\left[AA\right]e_{COX}\left(1+k_{ATP}\left[ATP\right]\right)$, first term in Equation 11) is higher due to the presence of enzyme $e_{COX}$ (Fig. 6C), and outflux of PGH_2_ ($g_{PGH_{2}}\left[PGH_{2}\right]+k_{PGD_{2}}\left[PGH_{2}\right]e_{Ptgds}+k_{PGE_{2}}\left[PGH_{2}\right]e_{Ptges}$, last three terms in Equation 11) is lower due to smaller degradation rate and negligible levels of $e_{Ptgds}$ (Fig. 6A) and $e_{Ptges}$ (Fig. 6B). However, for 2.5<t<10, PGH_2_ decreased because the influx of PGH_2_ reduced due to decreasing ATP and outflux of PGH_2_ increased as a result of rising $e_{Ptgds}$ (Fig. 6A) and $e_{Ptges}$ (Fig. 6B). For t>10 minutes, AA governed the temporal profile of PGH_2_. Similar arguments followed for the dynamics of EPA supplemented experiments for PGH_2_ and PGH_3_. The temporal evolution of PGD_2_/PGE_2_ and PGD_3_/PGE_3_ followed PGH_2_ and PGH_3_ dynamics, respectively. The present model adopts nonlinear kinetics for the metabolite rate balance, including control variables (*u*, *v*), nonlinearly modulate the rates. In contrast, the rate balances in Gupta *et al.* study were nonlinear due to the Michaelis-Menten form to model enzyme competition. Both models focus on mechanism; however, the conceptual difference lies in our model adopting the goal-seeking behavior of the system. The success of this model supported the notion of a goal-seeking nature during the inflammatory response.

## Conclusion

Our model provides insights into the overall behavior of cells, particularly their drive to achieve the biological goal of maximizing proinflammatory and antiinflammatory responses. It elucidates the underlying mechanism by formulating the cybernetic goal and enables the system to decide the best trajectory for accomplishing the defined biological and cybernetic goals. The trends for the control variables offered valuable insights into the dominance of metabolites, guiding the system to follow the appropriate underlying mechanism. In contrast, the previous study formulated the mechanistic form mathematically, directing the system to adopt an informed approach. Our model formulation was supported by the results of the F-test conducted during the model validation process (Table 1). The successful prediction of profiles for the leave-one-out-metabolite PGD_2_ cross-validation method, further affirmed accurate incorporation of mechanism within the cybernetic model. Furthermore, the cybernetic model developed can be adapted to study the unseen scenarios involving DHA supplementation. The reasonable fit observed for these cases further substantiates the efficacy of applying the cybernetic model with variations to novel datasets that adhere to similar underlying mechanisms. Future research directions could involve the formulation of a model that integrates AA and EPA/DHA metabolism with the dynamics of cytokines/chemokines, small signaling proteins that play a vital role in recruiting immune cells to specific tissues and are proinflammatory or antiinflammatory in nature. Their interactions and correlations with 2-series and 3-series PGs in disease contexts are well-established. Therefore, developing an integrated model could offer valuable insights into the mutual regulation of PGs and cytokines.

**Table 1:** The F-test results for all metabolites. For a significance value of 0.05, F_0.05_ (16, 32) = 0.46

Metabolite	F-test Value	Metabolite	F-test Value	Metabolite	F-test Value	Metabolite	F-test Value
PGE_2_	0.1105	dPGD_2_	0.0764	PGJ_2_	0.0618	PGD_3_	0.0713
PGD_2_	0.0202	dhkPGD_2_	0.299	PGE_3_	0.0294		

### Data availability

The experimental data are available in the publication by Norris and Dennis (59).

### Supplemental data

This article contains supplemental data.

## Conflict of interest

The authors declare that they have no conflicts of interest with the contents of this article.
