# Supplementary material for: Modeling enzyme competition in eicosanoid metabolism in macrophage cells using a cybernetic framework
Source: J Lipid Res. 2024 Oct 11;65(12):100666. doi: 10.1016/j.jlr.2024.100666 (PMC11728974; doi:10.1016/j.jlr.2024.100666)
Supplement: Supplementary Information [file mmc1.docx]

**Supplementary document**

**Supp. Figure 1:** The interpolated profiles of Arachidonic acid (AA) for Ctrl and EPA supplementation cases are shown.


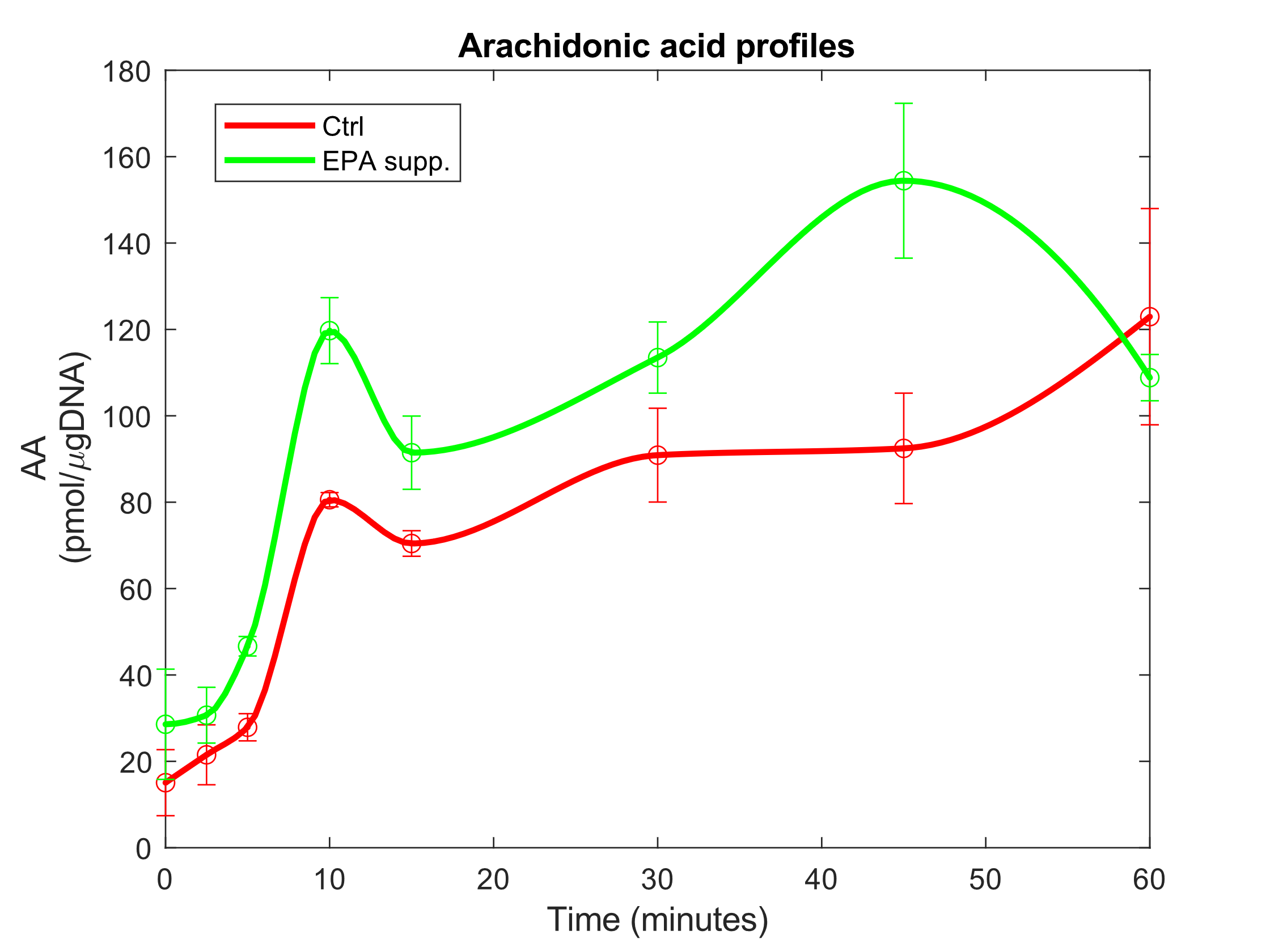


**Supp. Figure 2:** The interpolated profiles of Eicosapentanoic acid (EPA) for Ctrl and EPA supplementation cases are shown.


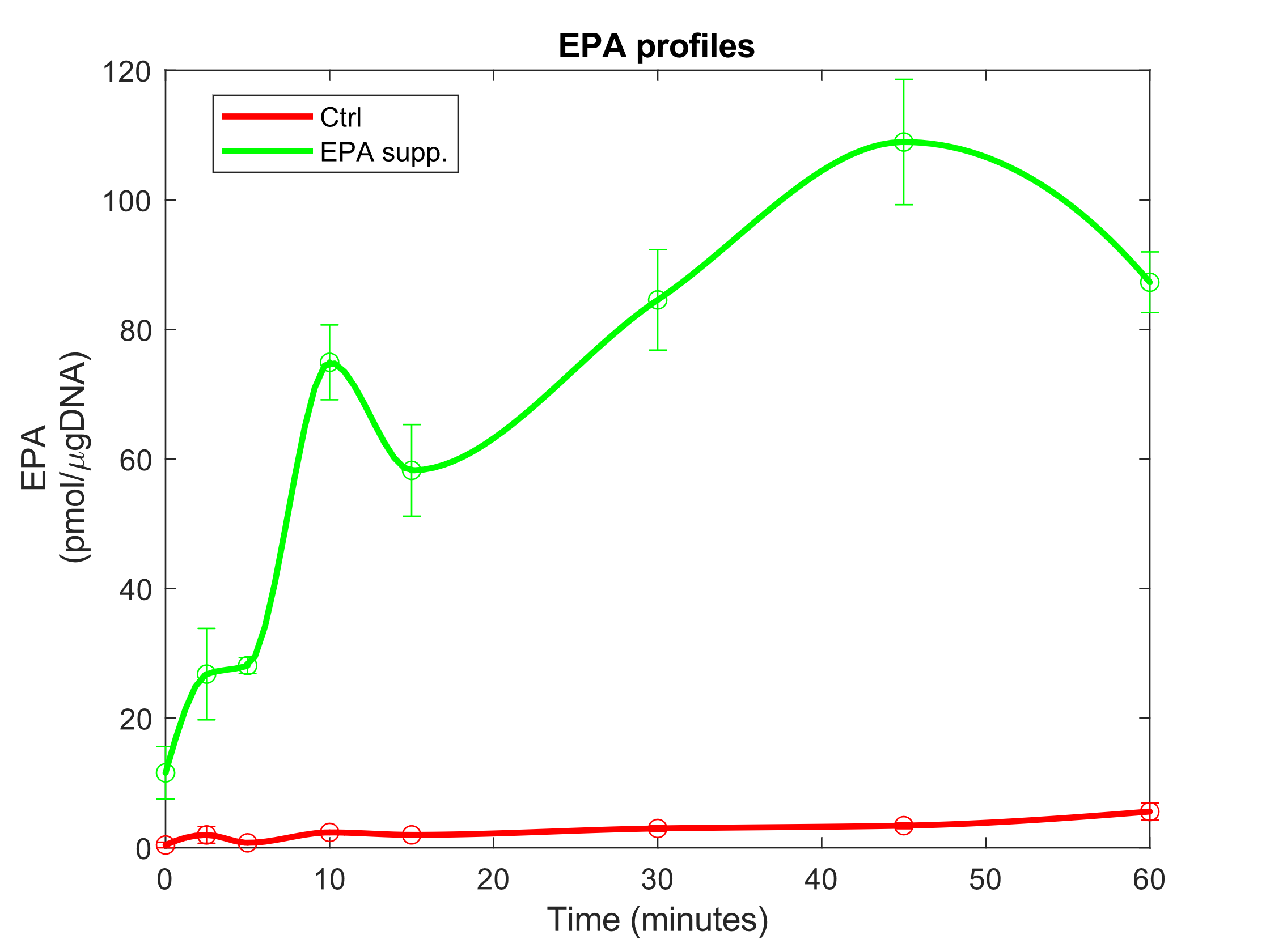


1. **Materials and Methods:**
   1. **Enzymatic reactions without cybernetic control variables**

The subsequent conversions of PGH_2_ and PGH_3_ are through the reactions in Eq. ((1) - (5)), which are enzymatic reactions without cybernetic control variables. The products PGD_2_ (Eq. (1)) and PGD_3_ (Eq. (2)) also share the enzyme $e_{Ptgds}$, and PGE_2_ (Eq. ( 3)) and PGE_3_ (Eq. (4)) utilize the enzyme $e_{Ptges}$. dhkPGD_2_ (Eq. (5)) is the downstream product of PGD_2_. There are no cybernetic variables associated with reactions (Eq. (1)-(5)).

$PGH_{2} +e_{Ptgds}\underset{\to}{k_{PGD_{2}}} PGD_{2}$ ( 1 )

$PGH_{3}+e_{Ptgds}\underset{\to}{k_{PGD_{3}}} PGD_{3}$ ( 2 )

$PGH_{2} +e_{Ptges}\underset{\to}{k_{PGE_{2}}} PGE_{2}$ ( 3 )

$PGH_{3}+e_{Ptges}\underset{\to}{k_{PGE_{3}}} PGE_{3}$ ( 4 )

$PGD_{2} +e_{dhkPGD_{2}}\underset{\to}{k_{dhkPGD_{2}}} dhkPGD_{2}$ ( 5 )

Eq. (6) is the generic differential equation for enzymatic reactions (Eq. (1) - ( 5 )) without cybernetic variables, where all constants have the same meaning as discussed before.

$\frac{d\left[ P_{i} \right]}{dt} = k_{P_{i}}\left[ S_{i} \right][e_{i}]- g_{P_{i}}\left[ P_{i} \right]- downstream fluxes$ ( 6 )

The ODEs (Eq. ( 7 )- ( 11 )) are written for specific metabolites using Eq. ( 18 ).

$\frac{d\left[ PGD_{2} \right]}{dt}=k_{PGD_{2}}\left[ PGH_{2} \right][e_{Ptgds}] - g_{PGD_{2}}[PGD_{2}] - k_{dPGD_{2}}[PGD_{2}] - k_{PGJ_{2}}[PGD_{2}] - k_{dhkPGD_{2}}[PGD_{2}]e_{dhkPGD_{2}}$ ( 7 )

The downstream fluxes for PGD_2_ (Eq. ( 7)) are due to its further conversions to dPGD_2_, PGJ_2_, and dhkPGD_2_ with corresponding rate constants as $k_{dPGD_{2}}$, $k_{PGJ_{2}}$, and $k_{dhkPGD_{2}}$. The downstream fluxes are zero for PGD_3_ (Eq. ( 8 )), PGE_2_ (Eq. ( 9 )), PGE_3_ (Eq. ( 10 )), and dhkPGD_2_ (Eq. ( 11 )). The kinetic rate constants for the production are $k_{PGD_{3}}$ for PGD_3_, $k_{PGE_{2}}$ for PGE_2_, $k_{PGE_{3}}$ for PGE_3_, and $k_{dhkPGD_{2}}$ for dhkPGD_2_. Their respective decay rate constants are $g_{PGD_{3}}$, $g_{PGE_{2}}$, $g_{PGE_{3}}$, and $g_{dhkPGD_{2}}$.

$\frac{d\left[ PGD_{3} \right]}{dt} = k_{PGD_{3}}\left[ PGH_{3} \right][e_{Ptgds}] - g_{PGD_{3}}\left[ PGD_{3} \right]$ ( 8 )

$\frac{d\left[ PGE_{2} \right]}{dt} = k_{PGE_{2}}[PGH_{2}]{[e}_{Ptges}] - g_{PGE_{2}}[PGE_{2}]$ ( 9 )

$\frac{d\left[ PGE_{3} \right]}{dt} =k_{PGE_{3}}\left[ PGH_{3} \right][e_{Ptges}] - g_{PGE_{3}}[PGE_{3}]$ ( 10 )

$\frac{d\left[ dhkPGD_{2} \right]}{dt} = k_{dhkPGD_{2}}\left[ PGD_{2} \right][e_{dhkPGD_{2}}] - g_{dhkPGD_{2}}[dhkPGD_{2}]$ ( 11 )

- 1. **Non-enzymatic reactions**

For non-enzymatic reactions, the enzyme is absent, and $k_{P_{j}}$ is the respective consistent rate constant. The reaction is written as Eq. ( 12). In this study, the substrate, $S_{j}$, for non-enzymatic reactions is PGD_2,_ and the products $P_{j}$ are PGJ_2_ (Eq. ( 13 )) and dPGJ_2_ (Eq. ( 14 )).

$S_{j}\underset{\to}{k_{P_{j}}} P_{j}$ ( 12 )

$PGD_{2} \underset{\to}{k_{PGJ_{2}}} PGJ_{2}$ ( 13 )

$PGD_{2} \underset{\to}{k_{dPGJ_{2}}} dPGJ_{2}$ ( 14 )

The ODE (Eq. ( 15 )) describes the kinetic rate balance for $P_{j}$, where $P_{j}$ is PGJ_2_ and dPGJ_2_ (Eq. ( 17 )).

$\frac{d\left[ P_{j} \right]}{dt} = k_{P_{j}}\left[ S_{j} \right]- g_{P_{j}}\left[ P_{j} \right]- downstream fluxes$ ( 15 )

The rate balances are specified for PGJ_2_ (Eq. ( 16 )) and dPGJ_2_ (Eq. ( 17 )). $k_{PGJ_{2}}$, $k_{dPGJ_{2}}$ are the rate constants and $g_{PGJ_{2}}$, $g_{dPGJ_{2}}$ are the degradation rates for PGJ_2_ and dPGJ_2_, respectively.

$\frac{d\left[ PGJ_{2} \right]}{dt} = k_{PGJ_{2}}[PGD_{2}] - g_{PGJ_{2}}[PGJ_{2}]$ ( 16 )

$\frac{d\left[ dPGJ_{2} \right]}{dt} = k_{dPGJ_{2}}[PGD2]- g_{dPGJ_{2}}[dPGJ_{2}]$ ( 17 )

- 1. **Enzyme balance in light of the cybernetic modeling**

There are no cybernetic variables associated with reactions other than PGH_2_ and PGH_3_ formations. The enzyme balances for $e_{Ptgds}$ (Eq. ( 18 )), $e_{Ptges}$ (Eq. ( 19 )), and $e_{dhkPGD_{2}}$ (Eq. ( 20 )) do not contain the control variables for enzyme synthesis. Their inductive rates depend on the substrate they act upon, but the maximum rate for Michaelis-Menten kinetics is not modulated. The substrates for $e_{Ptgds}$ and $e_{Ptges}$ are PGH_2_ and PGH_3_, and for $e_{dhkPGD_{2}}$, it is PGD_2_. For the enzyme balance of $e_{Ptgds}$ (Eq. ( 18 )), $k_{ePGD_{2}}$ and $k_{ePGD_{3}}$ are the maximum inducible rates due to the substrates PGH_2_ and PGH_3_, respectively. Similarly, for $e_{Ptges}$ (Eq. ( 19 )), $k_{ePGE_{2}}$ and $k_{ePGE_{3}}$ are the maximum inducible rates. $Km_{PGH_{2}}$and $K{m1}_{PGH_{2}}$ are the Michalis-Menten constants for PGH_2_ conversion into PGD_2_ and PGE_2_, $Km_{PGH_{3}}$and $K{m1}_{PGH_{3}}$ for PGH_3_ conversion into PGD_3_ and PGE_3_ and $Km_{PGD_{2}}$ for PGD_2_, respectively. The substrate for $e_{dhkPGD_{2}}$(Eq. ( 20 )) is PGD_2_ only. Hence, the inducible rate comprises substrate PGD_2_ alone, and $k_{edhkPGD_{2}}$ is the corresponding maximum inducible rate.

$\frac{d[e_{Ptgds}]}{dt} =\alpha+ k_{ePGD_{2}}\frac{\left[ PGH_{2} \right]}{Km_{PGH_{2}}+\left[ PGH_{2} \right]} +k_{ePGD_{3}}\frac{\left[ PGH_{3} \right]}{Km_{PGH_{3}}+[PGH_{3}]} -\beta[e_{Ptgds}]$ ( 18 )

$\frac{d[e_{Ptges}]}{dt} =\alpha+ k_{ePGE_{2}}\frac{\left[ PGH_{2} \right]}{K{m1}_{PGH_{2}}+[PGH_{2}]} + k_{ePGE_{3}}\frac{\left[ PGH_{3} \right]}{Km1_{PGH_{3}}+\left[ PGH_{3} \right]} -\beta{[e}_{Ptges}]$ ( 19 )

$\frac{d[e_{dhkPGD_{2}}]}{dt} =\alpha+ k_{edhkPGD_{2}}\frac{\left[ PGD_{2} \right]}{Km_{PGD_{2}}+[PGD_{2}]} -\beta[e_{dhkPGD_{2}}]$ ( 20 )

- 1. **Prediction for unseen cases of control and DHA supplementation**

The enzymes $e_{Ptgds}$ (Eq. ( 21 )) and $e_{Ptges}$ (Eq. ( 22 )) catalyze the conversion of the substrate PGH_2_ only. The enzyme balance for $e_{dhkPGD_{2}}$ (Eq. ( 20 )) remains the same. We simulated the system using the above-mentioned simulation strategy and determined the parameters.

$\frac{d[e_{Ptgds}]}{dt}=\alpha+ k_{ePGD_{2}}\frac{\left[ PGH_{2} \right]}{Km_{PGH_{2}}+\left[ PGH_{2} \right]} -\beta[e_{Ptgds}]$ ( 21 )

$\frac{d[e_{Ptges}]}{dt} =\alpha+ k_{ePGE_{2}}\frac{\left[ PGH_{2} \right]}{K{m1}_{PGH_{2}}+[PGH_{2}]} -\beta[e_{Ptges}]$ ( 22 )

1. **Parameters**

**Supp. Table 1: EPA-supplemented experimental condition**

| k_PGD2_ | 1.278436 | K_mEPA_ | 99.70059 | e_dhkPGD20_ | 0.981312 |
| --- | --- | --- | --- | --- | --- |
| k_PGD3_ | 1.719189 | K_mPG2_ | 0.32648 | K_mPG21_ | 2.626164 |
| k_PGE2_ | 2.899214 | K_mPG3_ | 0.919713 | K_mPG31_ | 5.767694 |
| k_PGE3_ | 0.304523 | alpha | 0.001635 |  |  |
| k_dPGD2_ | 0.005711 | beta | 0.314142 |  |  |
| k_PGJ2_ | 0.020748 | k_ePGD2_ | 0.862729 |  |  |
| k_dhkPGD2_ | 0.109803 | k_ePGD3_ | 1.006691 |  |  |
| k_PGH2_ | 2.879705 | k_ePGE2_ | 0.326489 |  |  |
| k_PGH3_ | 7.623249 | k_ePGE3_ | 0.849705 |  |  |
| g_PGD2_ | 8.81E-06 | k_ePGH2_ | 0.015573 |  |  |
| g_PGD3_ | 11.1593 | k_ePGH3_ | 0.000485 |  |  |
| g_PGE2_ | 0.403185 | PGH20 | 0.089779 |  |  |
| g_PGE3_ | 2.023706 | e_PGD20_ | 0.003302 |  |  |
| g_dPGD2_ | 0.321424 | e_PGE20_ | 0.101615 |  |  |
| g_PGJ2_ | 0.619829 | e_PGH20_ | 0.079287 |  |  |
| g_dhkPGD2_ | 9.829051 | k_d_ | 3.162278 |  |  |
| g_PGH2_ | 1.55E-09 | k_ATP_ | 0.900343 |  |  |
| g_PGH3_ | 1.28E-10 | k_edhkPGD2_ | 0.25657 |  |  |
| K_mAA_ | 27.85205 | K_mPGD2_ | 14.38684 |  |  |

**Supp. Table 2: DHA-supplemented**

| k_PGD2_ | 0.929663 | K_mEPA_ | 0.002804 | k_ATP_ | 4.104023 |
| --- | --- | --- | --- | --- | --- |
| k_PGE2_ | 6.958102 | K_mPG2_ | 0.212536 | k_edhkPGD2_ | 0.001085 |
| k_dPGD2_ | 0.015974 | K_mPG3_ | 0.526932 | K_mPGD2_ | 23.10859 |
| k_PGJ2_ | 0.053077 | alpha | 2.37E-05 | e_dhkPGD20_ | 0.233696 |
| k_dhkPGD2_ | 0.407501 | beta | 1.883227 | K_mPG21_ | 0.362056 |
| k_PGH2_ | 0.441071 | k_ePGD2_ | 6.415827 |  |  |
| k_P_ | 4.414171 | k_ePGE2_ | 0.121797 |  |  |
| g_PGD2_ | 0.018665 | k_ePGH2_ | 0.239501 |  |  |
| g_PGE2_ | 0.065136 | k_eP_ | 0.256583 |  |  |
| g_dPGD2_ | 0.758414 | PGH20 | 0.185658 |  |  |
| g_PGJ2_ | 1.232199 | P_D0_ | 0.002437 |  |  |
| g_dhkPGD2_ | 1.78E-08 | e_PGD20_ | 0.0015 |  |  |
| g_PGH2_ | 3.17E-11 | e_PGE20_ | 1.079057 |  |  |
| g_P_ | 7.758655 | e_PGH20_ | 0.192904 |  |  |
| K_mAA_ | 0.054413 | k_d_ | 3.162278 |  |  |

**Supp. Table 3: Leave-One-Out (LOO) PGD_2_ scenario**

| k_PGD2_ | 0.429346 | K_mAA_ | 2.197362 | k_edhkPGD2_ | 0.135178 |
| --- | --- | --- | --- | --- | --- |
| k_PGD3_ | 9.898084 | K_mEPA_ | 45.72588 | K_mPGD2_ | 4.845828 |
| k_PGE2_ | 5.959712 | K_mPG2_ | 0.18986 | e_dhkPGD20_ | 0.001013 |
| k_PGE3_ | 1.257599 | K_mPG3_ | 0.157196 | K_mPG21_ | 0.613282 |
| k_dPGD2_ | 0.013564 | alpha | 9E-05 | K_mPG31_ | 7.054066 |
| k_PGJ2_ | 0.028827 | beta | 0.812224 |  |  |
| k_dhkPGD2_ | 0.15122 | k_ePGD2_ | 0.971476 |  |  |
| k_PGH2_ | 9.177064 | k_ePGD3_ | 0.188396 |  |  |
| k_PGH3_ | 20.62475 | k_ePGE2_ | 0.596773 |  |  |
| g_PGD2_ | 0.025056 | k_ePGE3_ | 1.801478 |  |  |
| g_PGD3_ | 3.963466 | k_ePGH2_ | 0.005552 |  |  |
| g_PGE2_ | 0.336676 | k_ePGH3_ | 0.000449 |  |  |
| g_PGE3_ | 0.9964 | PGH20 | 0.00127 |  |  |
| g_dPGD2_ | 0.051716 | e_PGD20_ | 0.662491 |  |  |
| g_PGJ2_ | 0.059246 | e_PGE20_ | 0.363785 |  |  |
| g_dhkPGD2_ | 0.116862 | e_PGH20_ | 0.050516 |  |  |
| g_PGH2_ | 1.939932 | k_d_ | 3.162194 |  |  |
| g_PGH3_ | 0.519852 | k_ATP_ | 0.149468 |  |  |

1. **Model Validation**

**3.1 F-test:**

$F=\frac{\frac{\left( \sum_{j=1}^{nt} \left( Y_{j}^{trt}-\bar{X}_{j}^{trt} \right)^{2}+\sum_{j=1}^{nt} \left( Y_{j}^{ctrl}-\bar{X}_{j}^{ctrl} \right)^{2} \right)}{(ne\times nt)}}{\frac{\left( \sum_{j=1}^{nt} \sum_{i=1}^{nr} \left( X_{ij}^{trt}-\bar{X}_{j}^{trt} \right)^{2}+\sum_{j=1}^{nt} \sum_{i=1}^{nr} \left( X_{ij}^{ctrl}-\bar{X}_{j}^{ctrl} \right)^{2} \right)}{(ne\times nt\times(nr-1))}}$ ( 23 )

where $X_{j}$,  $\bar{X}_{j}$, and $Y_{j}$ denote the experimental data, mean experimental data, and simulated (fitted) data at time point *j*, respectively. *nr* is the number of replicates (*nr* = 3, indexed as i), *nt* is the number of time points (*nt* = 8, indexed as *j*). *ne* is the number of experimental conditions used, and trt and ctrl are treatment and control groups, respectively (*ne* = 2). The degrees of freedom for determining the F-distribution are *df*_1_ = $\left( ne\times nt \right)$ and *df*_2_ = $(ne\times nt\times(nr-1))$.

**3.2 Kinetic balance equations for DHA supplemented case:**

$\frac{d\left[ PGH_{3} \right]}{dt} = k_{PGH_{3}}v_{PGH_{3}}\left[ DHA \right][e_{COX}](1+k_{ATP}[ATP]) - g_{PGH_{3}}[PGH_{3}]$ ( 24 )

$\frac{d[e_{Ptgds}]}{dt}=\alpha+ k_{ePGD_{2}}\frac{\left[ PGH_{2} \right]}{Km_{PGH_{2}}+\left[ PGH_{2} \right]} -\beta[e_{Ptgds}]$ ( 25 )

$\frac{d[e_{Ptges}]}{dt} =\alpha+ k_{ePGE_{2}}\frac{\left[ PGH_{2} \right]}{K{m1}_{PGH_{2}}+[PGH_{2}]} -\beta[e_{Ptges}]$ ( 26 )

$\frac{d[e_{dhkPGD_{2}}]}{dt} =\alpha+ k_{edhkPGD_{2}}\frac{\left[ PGD_{2} \right]}{Km_{PGD_{2}}+[PGD_{2}]} -\beta[e_{dhkPGD_{2}}]$ ( 27 )
